# Order among chaos: Cross-linguistic differences and developmental trajectories in pseudoword reading aloud using pronunciation Entropy

**DOI:** 10.1371/journal.pone.0251629

**Published:** 2021-05-19

**Authors:** Elisabetta De Simone, Elisabeth Beyersmann, Claudio Mulatti, Jonathan Mirault, Xenia Schmalz

**Affiliations:** 1 Department of Child and Adolescent Psychiatry, Psychosomatics and Psychotherapy, University Hospital, LMU Munich, Munich, Germany; 2 Department of Cognitive Science, Macquarie University Centre of Reading, Sydney, Australia; 3 Department of Psychology and Cognitive Science, Università degli studi di Trento, Trento, Italy; 4 Aix-Marseille University and Centre National de la Recherche Scientifique, Marseille, France; Universiteit van Amsterdam, NETHERLANDS

## Abstract

In this work we propose the use of Entropy to measure variability in pronunciations in pseudowords reading aloud: pseudowords where participants give many different pronunciations receive higher Entropy values. Monolingual adults, monolingual children, and bilingual children proficient in different European languages varying in orthographic depth were tested. We predicted that Entropy values will increase with increasing orthographic depth. Moreover, higher Entropy was expected for younger than older children, as reading experience improves the knowledge of grapheme-phoneme correspondences (GPCs). We also tested if interference from a second language would lead to higher Entropy. Results show that orthographic depth affects Entropy, but only when the items are not strictly matched across languages. We also found that Entropy decreases across age, suggesting that GPC knowledge becomes refined throughout grades 2-4. We found no differences between bilingual and monolingual children. Our results indicate that item characteristics play a fundamental role in pseudoword pronunciation variability, that reading experience is associated with reduced variability in responses, and that in bilinguals’ knowledge of a second orthography does not seem to interfere with pseudoword reading aloud.

## Introduction

It is common practice in reading research to use pseudowords in order test participants’ ability to use grapheme-phoneme correspondences (GPCs) to correctly retrieve sound from print [1]. This ability is considered fundamental to learning to read: since children at the beginning of reading acquisition do not have a large sight vocabulary, they need to more heavily on their knowledge of letter-sound correspondences to assemble the correct pronunciation, a process known as phonological decoding [2].

Pseudowords have received a great deal of attention in this field. Pseudowords are graphotactically legal stimuli with plausible pronunciations [3]. Their importance lies in their helpfulness in predicting poor reading skills: studies have shown that dyslexic readers perform worse than their non-impaired peers on pseudowords reading aloud tasks [4, 5]. Pseudowords are usually assessed by calculating reaction times (the time between stimulus onset and voice onset) and reading accuracy (the number of errors that participants make while reading). Concerning reaction times, two assumptions underlie its use for inference: Firstly, they have to assume that if a participant is taking more time in naming a particular item, it means that item is more difficult than others. Secondly, the researcher has to hypothesize about features of that particular item that make it difficult to name. For example, when 100 participants read aloud two pseudowords, “rop” and “wap”, they might have faster reaction times to the former than to the latter. With this finding, we can calculate differences, on the linguistic level, between these two pseudowords (e.g. in terms of vowel consistency, orthographic neighborhood or letter bigram frequency). This would allow for indirect inferences about which linguistic characteristics affect reading aloud processes, which would, in turn, allow us to hypothesize a cognitive structure that would explain why this particular characteristic should affect reading processes. The transcribed responses of the participants give more direct information about the cognitive processes [6–8]. For example, for the two pseudowords above, participants might pronounce the former consistently as /ɹɔp/, and for the latter, some participants might pronounce the pseudoword as /wæp/ or as /wɔp/. This is more direct evidence that consistency (i.e., the presence of more than one possible pronunciation for the letter cluster *wa*, “in wasp” versus “wax”) affects reading aloud processes.

As for accuracy, since pseudowords do not have conventional pronunciations, it is difficult to decide whether they are pronounced correctly or not [7, 9]. Often, faced with the variety of responses participants give, researchers need to arbitrarily decide whether a pseudoword is correctly read by analyzing all the plausible pronunciations that they think it could have [9–11]. Even if a given software is used to score accuracy, decisions need to be made concerning response accuracy. For example, if we accept any pronunciation as correct whenever there is at least one instance of the grapheme-phoneme correspondence in the language, we would consider, the pronunciation /jɔn/ for the English pseudoword <yan> as correct, although, intuitively, most English native speakers would consider this pronunciation incorrect, because it corresponds to the vowel pronunciation of the word <yacht>.

With this in mind, we aim to investigate the number and kind of different pronunciations participants give, an information that is not captured by only scoring the answers as correct and incorrect [6–8]: The quantification of response variability to a given pseudoword may be a more sensitive measure of pseudoword reading aloud performance, since it does not involve any kind of arbitrary decisions from the researchers. Of course, the variability of responses and accuracy may be correlated: If participants give many different pronunciations to a given pseudoword, by definition, the variability will be high for this item. This also implies that any scoring scheme would likely mark more responses as incorrect.

Considering this, our study’s goal was to test an alternative variable, namely pseudoword reading aloud Entropy, as a way to quantifying participants’ pseudoword reading aloud performances [12]. This approach has the advantage that rather than making decisions about whether a given pronunciation is incorrect, we can include and analyze all responses.

### Pseudowords pronunciation Entropy

Entropy is a concept first introduced by Shannon’s Information Theory [13], which can be defined as the degree of chaos within a closed system. Earlier studies in psycholinguistic research used Entropy as a measure to investigate processing difficulty in sentence comprehension [14], quantify orthographic transparency in different orthographies (using word onsets: [15–17], using mono-syllabic words: [18], using whole words: [19]), and to assess variability in responses to disyllabic English pseudowords [20] as well as diversity in vowel pronunciation in German and English children reading aloud pseudowords [12].

In the present study, we use Entropy to calculate the variability of responses to both monosyllabic and multisyllabic pseudowords. This considering, we focus in this study on the following three aspects:

1. Orthographic depth, by investigating orthographies varying in depth (English, German, French, Italian);
2. Age (adults and children) and grade (2, 3, 4, for monolingual German children):
3. r Bilingualism (comparing bilingual English-German children, reading German items, with monolingual German children)

Entropy values are calculated as follows: the more alternative pronunciations a given pseudoword has, the bigger its Entropy value is. Since Entropy focuses on the whole pseudoword pronunciation, Entropy values are not affected by the readers’ strategy to retrieve sound from larger (morphemes, bodies) or smaller embedded reading units (letters, graphemes). For each pseudoword, we have the transcription of each participant reading this particular item. Entropy is calculated, for each item, by taking the percentage of each type of response, multiplying it by its logarithm, and summing the resulting value for all possible pronunciations of this item. This process is described in the formula:

$$\begin{array}{c} H_{j}=-\sum_{i=1}^{N}p\left(i|j\right)\cdot \log_{2}p(i|j) \end{array}$$

where *p*(*i*|*j*) refers to the percentage of responses *i* for item *j*, where *N* is the number of different pronunciations provided across the participants. Negative numbers were converted into positive numbers (because the logarithm of a proportion, i.e., a number between 0 and 1, is always negative) for easier interpretability, by multiplying the summed Entropy value for each item *j* by -1. An example of how Entropy is calculated for a specific item can be found in Table 1.

**Table 1:** How to calculate Entropy from participants pronunciations for the pseudoword <wap>.

Pronunciations	Proportion	Proportion * Log
(7) wæp	7/(7 + 9 + 1) = 0.41	0.41 * log_2_(0.41) = 0.53
(9) wɔp	9/(7 + 9 + 1) = 0.53	0.53 * log_2_(0.53) = 0.49
(1) wælp	1/(7 + 9 + 1) = 0.06	0.06 * log_2_(0.06) = 0.24
		Entropy
		1.26

Note: In the Proportion * Log column we multiplied the numbers by -1 for easier interpretability

When participants provided the same pronunciation for a given pseudoword, the Entropy value of that item was zero, because log1 = 0. Higher Entropy values (*H* > 0) instead resulted from participants giving different pronunciations, and as the distribution of multiple pronunciations approaches equiprobability. This formula allows us to focus on item-level differences, that is, to calculate Entropy per item, while for subject-level performances, we average across participants.

To summarize, Entropy is defined here as the number of different pronunciations that participants give to the same pseudoword (pseudoword pronunciation variability). For example, in a sample of five participants, Participants 1 and 3 could read the pseudoword <wap> as /wæp/; Participant 2, instead, would read the item as /wɔp/, while Participants 4 and 5 would agree on a yet different pronunciation: /wælp/. These different choices would increase the Entropy value associated with the pseudoword <wap>, calculated as seen in Table 1. However, the same five participants could agree on the pronunciation of another pseudoword: for example, all of them could read <drell> as /drel/. In this case, the Entropy value of <drell> would be equal to zero. As we will discuss below, there are reasons to think that pseudoword pronunciation variability (Entropy) may vary according to Language, Bilingualism and Age.

### Orthographic depth

As for orthography, the relationship between letters and sounds can affect Entropy. The closeness of this relationship is referred to as orthographic depth, and is traditionally described as a continuum [21]. For example, on the shallow end of the continuum are orthographies like Finnish or Italian, where one letter typically corresponds to one sound (i.e, <i> only maps to /i/), while on the deep end are orthographies with a high degree of inconsistency between its letters and sounds (i.e. in the word “gist” <g> is read /ʤ/, but the grapheme itself could be read /g/ as well), like English [22].

Shallow orthographies are easier to read and learn [21, 23–26] because of the straightforward mapping between graphemes and phonemes. Italian and German, for instance, are considered to have shallow orthographies [21], therefore we expected that the Entropy value of pseudowords read by our Italian and German participants will be very low, because the consistent correspondences between graphemes are phonemes will lead to none or very few possible alternative pronunciations (e.g., in Italian, <fulm> can be only read /fulm/ because all the letters in that pseudoword have only one phoneme corresponding to them, leading to only one possible pronunciation). Consequently, since the pseudowords do not have many different pronunciations, their Entropy was also expected to be low.

On the opposite end of the continuum are deep orthographies like English. Children learning to read in deep orthographies have been found to take longer to learn the correspondences between letters and sounds, because of their inconsistent and unpredictable relationships (the same grapheme <i>, found in words like <kit> and <pint> will be read /ɪ/ in the first case and /aɪ/ in the second). As a result, it takes longer to acquire the ability to read accurately [21, 23–25]. We expect that the pseudoword Entropy value for English-speaking children and adults will be the highest, because letters are normally associated with more than one sound, leading to multiple alternative pronunciations (for the pseudoword <sind> can be read /s nd/ or /sa nd/). For this reason, we predicted higher respoonse variability in English-speaking children than in adults (because of their scarcer knowledge of GPCs); and higher response variability in English-speaking participants than in French-, Italian- and German-speaking children, who are learning to associate graphemes to phonemes in more consistent and transparent orthographies.

### Complexity and unpredictability

More recent work suggests that orthographic depth should not be seen as a single continuum, but rather as a multidimensional space [27–29]. Even within Europe, orthographies differ on many aspects which are difficult to condense into a single construct. While inconsistency of the print-to-speech correspondences has always been central to the concept of orthographic depth, the study from Schmalz, Marinus, Coltheart and Castles [28] showed that, across orthographies, inconsistency can result either from “complexity” or “unpredictability” which, according to models of reading, should have differential effects on cognitive processes underlying reading and reading acquisition.

Complexity, on the one hand, can lead to inconsistency on the level of letters or graphemes due to the presence of multiletter-correspondences (<aw> → /ɔ:/; this is a complex correspondence because the reading of the individual letters will not give the exact pronunciation), or due to the presence of context-sensitive correspondences (<g[i]> -> /ʤ/; <g[a]> → /g/) or from both (<ch[r]> → /k/; <ch[i]> -> /tʃ/). The French word “ciseaux”, for example, contains three complex correspondences: the context-sensitive rule dictates that <c[i]> is read /s/, while the multiletter grapheme <au> corresponds to /o/ and a position correspondence dictates that the plural morpheme <x> is silent because of its position at the end of the word. Nonetheless, even if there are three different context correspondences, the pronunciation is entirely predictable. Unpredictability, on the other hand, refers to the degree to which the reading system is capable of correctly translating written words into their phonological equivalents [28]. The pronunciation of the word “yacht”, for example, is unpredictable, because this word cannot be read correctly without the reader having encountered it before.

Within languages, complexity and unpredictability are correlated. This makes it difficult to dissociate between them. For example, in the English orthography it can be hard to dissociate complexity from unpredictability, as for example in the words “range” and “flange”. English phonotactics correspondences state that if an <a> is to be found before the ending <nge> then it should be read as /eɪ/, as in “range” (/reɪnʤ/). However, “flange” is not read /fleɪnʤ/, but /flænʤ/. In this case there is a grapheme which is read differently while being in the same context: in “range” a complex correspondence is applied (a + nge), while in “flange” a simple grapheme-phoneme correspondence is used (<a> is read /æ/). Thus, complex context-sensitive correspondence alone cannot predict how we should read <a>, and readers are often unsure about which strategy is to be applied (context-sensitive or simple GPCs?). Instances like the case we described are not rare, and they make English orthography both highly complex and unpredictable.

The French orthography, on the contrary, is high in complexity, but low in unpredictability. On the one hand. it presents many complex correspondences, caused by multiletter and context-sensitive graphemes (respectively like <au> and <c>). On the other hand, these correspondences are mostly predictable (<au> will be always only read as /o/, while <c> will always be read /s/ before <i, e> and /k/ before <a, o, u>).

Considering the relation between complexity and unpredictability, in the current study we will look at languages that are simple and predictable (Italian and, to a lesser degree, German), complex and predictable (French) and complex and unpredictable (English), in order to investigate the possibility that these features may differentially affect Entropy.

### Bilingualism

Another factor that may influence pseudowords pronunciation Entropy is bilingualism. Two scenarios are possible: when told to read pseudowords in Language A, individuals could show interference from Language B, by associating phonemes of Language B to graphemes of Language A. For example, English/German bilingual may read a German pseudoword like “moch” as /moʦ/ instead of /moχ/, because the grapheme <ch> is read differently in English. Similarly Treiman, Kessler and Evans [30] found interferences from French to English<c>and<g>pronunciation in English-speaking students who just started learning French. Thus, a grapheme-phoneme correspondence from Language B that interferes with reading Language A, may increase Entropy for bilingual individuals compared to monolingual individuals.

The second scenario goes in the opposite direction. Studies have shown that bilingualism improves metalinguistic awareness, that is the ability “to think about and reflect upon the nature and functions of language” [31]. Metalinguistic awareness refers to different aspects of language, as for example word awareness and phonological awareness. Moreover, results from Yelland, Pollard and Mercuri [43] show that this improved metalinguistic awareness in bilingual children also enhances reading skills, at least in regards to word recognition. Consequently, there are reasons to believe that bilingual children’s metalinguistic awareness could improve the overall understanding and sensitivity to GPCs, especially if one of the languages is more transparent than the other. For example, the prior learning of one consistent orthography could help understand the mechanisms underlying the GPCs in the other language, because children already have experience with the dynamics of associating letters to sounds, thus producing a facilitatory effect on the other language.

### Aim and hypothesis

Our study’s goal was to evaluate the use of Entropy (H) in participants’ pseudoword reading aloud responses. Although Entropy has already been used to measure the diversity of vowel pronunciations in German and English children reading aloud pseudowords across grades [12], alternative pronunciations of disyllabic pseudowords in English [20], we are the first, to our knowledge, to use it to compare individual responses to both mono-syllabic and multi-syllabic pseudowords across age (primary school children and adults) and languages (shallow and deep orthographies), including a consideration for bilingualism (in children).

In Experiment 1, we re-analyze novel and published pseudoword reading aloud data from different languages (Italian, German and English) which are on different points along the orthographic depth continuum. In Experiment 2, 3 and 4 we report new data from different age groups. According to the Orthographic Depth Hypothesis [32], we expect that readers of shallow orthographies (like Italian, and, to a lesser degree, German) will be associated overall with low Entropy values, because the very predictable and consistent GPC of their orthography should prevent the possibility of many different alternative pronunciations for pseudowords.

Readers of deep orthographies (like English) will be more likely to be associated with higher Entropy values: this is because in deep orthographies different phonemes can be assigned to one grapheme, which translates to the higher probability that the same pseudoword will be read differently, depending on which phonemes the individual will decide to assign to the graphemes contained in the given pseudoword. A second prediction concerns age.

Adults, as well as children from different grades (2, 3 and 4), participated in this study. We expect that overall children would show a greater variability in responses in all language groups compared to adults (exception made for Italians, for which we only have data from children), because their reading skills development is still on-going, that is, their knowledge of graphemes-phonemes mapping is still incomplete. Hence, children may assign a greater number of phonemes to a given grapheme, because of a greater uncertainty regarding GPCs. A direct comparison will be made among monolingual German children in grade 2, 3 and 4 to investigate whether younger children show greater response variability in responses compared to older children. Overall, we expected that grade 2 children’s responses to show higher Entropy values compared to grade 3 and grade 4 children, and grade 3 children to show higher Entropy values compared to grade 4 children.

With respect to bilingualism, as discussed earlier in the introduction, we believe that two outcomes may be possible: If it is true that grapheme-phoneme correspondences from one language interfere with the reading of the other language, we would expect that higher Entropy values will be reported in bilingual children’s responses. However, if it is true that enhanced metalinguistic awareness in bilinguals lead to enhanced reading skills compared to monolinguals, we would expect that, on the contrary, bilingual children responses will be associated with lower Entropy values compared to monolinguals.

## Experiment 1: Entropy in German and English adults reading matched pseudowords

In the first experiment, we re-analyzed pseudoword reading aloud data from a previously published study [33]. This study aimed to compare the nature of sublexical processing in English and German. The items were chosen such that they were matched on orthographic characteristics, such as the number of letters and orthographic neighborhood. In the published study, only RT data were analyzed. Here, we are extending the published data by providing new insights into the role of Entropy on pseudoword reading in German and English.

### Methods

#### Participants

German (n = 19) and Australian (n = 48) adults participated in this study. All were staff or students at universities in Germany and Australia, respectively, and received course credit or a small monetary compensation for their participation. The procedure was approved by the ethics committees of both Macquarie’s University, Australia (Macquarie University Faculty of Human Sciences (FHS) Ethics Committee) and Ludwig-Maximilian University, Germany (Ethikkommission bei der Medizinischen Fakultät der LMU München).

#### Materials

Participants read aloud pseudowords in their respective language, which were chosen in respect to the size of their body-neighborhood (see [34]). The size of the body-neighborhood (body-N) for all items was measured thanks to the CELEX database, which is available for both German and English. In the original experiment, participants read aloud both words and pseudowords (in their respective languages) varying in body-N while being matched across body-N condition on length and orthographic neighborhood. Here, we analyze only the pseudoword data. The pseudowords were monosyllabic and matched on the number of letters and orthographic neighborhood [35], as well as on body-neighborhood [34]. Moreover, all items had consistent bodies (i.e., while the number of body-neighbors was manipulated, all body-neighbors had the same pronunciation). Altogether, there were 90 English and 90 German pseudowords, half of which contain high-frequency bodies and the other half contain low-frequency bodies.

#### Procedure

Each participant was tested individually in a dimly lit, sound-proof testing booth. Each item was shown on the screen for 5 seconds or until the voicekey was triggered, in random order. The items were presented, one at a time, using the software DMDX [55], which created audio recordings for each participants and each item. Here, we analyze only the pseudoword reading aloud responses. A native speaker of each language transcribed the participants’ responses from the audiofiles previously recorded and a scorers who had received training in the phonology of the respective language scored the pronunciation accuracy. Both scorers were told to follow a lenient marking criterion, that is, all legally possible grapheme-phoneme relations (including context-inappropriate relations) were considered correct [23, 36, 37]. We then calculated the Entropy, for each pseudoword, using the formula described in the introduction and analyzed the data using the statistical environment software R [38]. Afterwards, as an additional analysis, we accounted for non-plausible pronunciations and random noise (meaningless misreadings, such as “dolt” read as /bolt/) by calculating Levenshtein distance [39] from the most common reading to a given pseudowords and all other alternative readings. We did a normalization of the distances obtained (by dividing the distance by the number of phonemes) so that it could be compared one to another. Since our shorter items counted three letters, we decided to exclude all pronunciations whose Levenshtein distance was higher than 0.334. With the resulting, diminished datasets, we then re-calculated Entropy and statistical tests (this re-analysis will be referred from now on as “pronunciation plausibility analysis”). The Python scripts which we used to calculate the Entropy values, as well as supplementary files, can be found here: https://osf.io/94wjx/.

### Results and discussion

Non-responses (1 trial from the German data, 6 trials from the English data) were excluded before calculating the Entropy. For German, the median of the Entropy value, across all items, was 0.48 (min = 0, max = 2.21), and for English, the median Entropy was 0.39 (min = 0, max = 1.96).

As the Entropy measure is still relatively new to the field of pseudoword reading, the first question we asked was whether Entropy for each item depends on random or systematic factors. As the English sample was larger than the German sample, we randomly split the English sample 25 times into two groups of 24 participants each, and calculated the item-level Entropy for each item for the two different sub-samples. The mean of the correlations between the fifty sub-samples was 0.89, with a standard deviation of 0.02. All of the correlations were significant *r*(90) = *p* < 0.001.

The second question was if and how Entropy correlated with accuracy. Two scorers scored English pronunciation accuracy, while one scorer scored German pronunciation accuracy. We then calculated a correlation matrix between Entropy, accuracy, number of answers and percentage of the most common responses for both groups. Table 2 shows the results for English speaking participants, while Table 3 shows the results for German speaking participants. The agreement between scorers was calculated with Cohen’s kapp to measure inter-rater reliability [40]. Results show that, for the English data, the scorers were in a moderate agreement (*k* = 0.57).

**Table 2:** Intercorrelations for English-speaking participants (Exp 1).

Measure	1	2	3	4	5
1. Entropy	-	.26*	.04	.73*	-.86*
2. acc_s2	.26*	-	.29*	.17	-.21*
3. acc_s1	.04	.29*	-	.02	.03
4. n_sw	.73*	.17	.02	-	-.75*
5. perc	-.86*	-.21*	.03	-.75*	-
n = 90					

Note: n_asw = number of different pronunciations per pseudowords, acc_s1 and acc_s2 = accuracy scored by scorer 1 and 2, perc = percentage of the most common response,

* = significant result.

**Table 3:** Intercorrelations for German-speaking participants (Exp 1).

Measure	1	2	3	4
1. Entropy	-	-.34*	.92*	-.94*
2. acc	-.34*	-	-.47*	.20
3. n_asw	.92*	-.47*	-	-.76*
4. perc	-.94*	.20	-.76*	-
n = 90				

Note: n_asw = number of different pronunciations per pseudowords, acc = scored accuracy, perc = percentage of the most common response,

* = significant result.

Entropy was weakly correlated with accuracy, in a significant fashion for scorer 2: *r* = 0.26, *p* < 0.05 but not for scorer 1: *r* = 0.04, *p* = 0.70. This result was unexpected: Entropy was expecteed to be correlated negatively with accuracy, because it was calculated based on the number of pronunciations. This means that scorers were more likely to accept several alternative pronunciations as correct for English than for German, with the latter showing a negative correlation (*r* = −0.34, *p* < 0.05).

As expected, we found a significant positive correlation with the number of pronunciations per English pseudowords: *r* = 0.73, *p* < 0.001, showing that items with a high Entropy received more different pronunciations than items with a low Entropy, and a significant negative correlation with the percentage of the most common pronunciation (*r* = −0.86, *p* < 0.001). In German participants, Entropy negatively correlated with the accuracy scoring (*r* = −0.34, *p* < 0.05). This is more in line with what we would expect: as accuracy is high, Entropy is naturally low. However, since we could not recruit a second scorer for the German data, the reliability of this correlation remains to be seen. For the other measures, Entropy correlated positively, with the number of pronunciations (*r* = 0.92, *p* < 0.001) and negatively with the percentage of the most common response (*r* = −0.94, *p* < 0.001).

**Fig 1:**
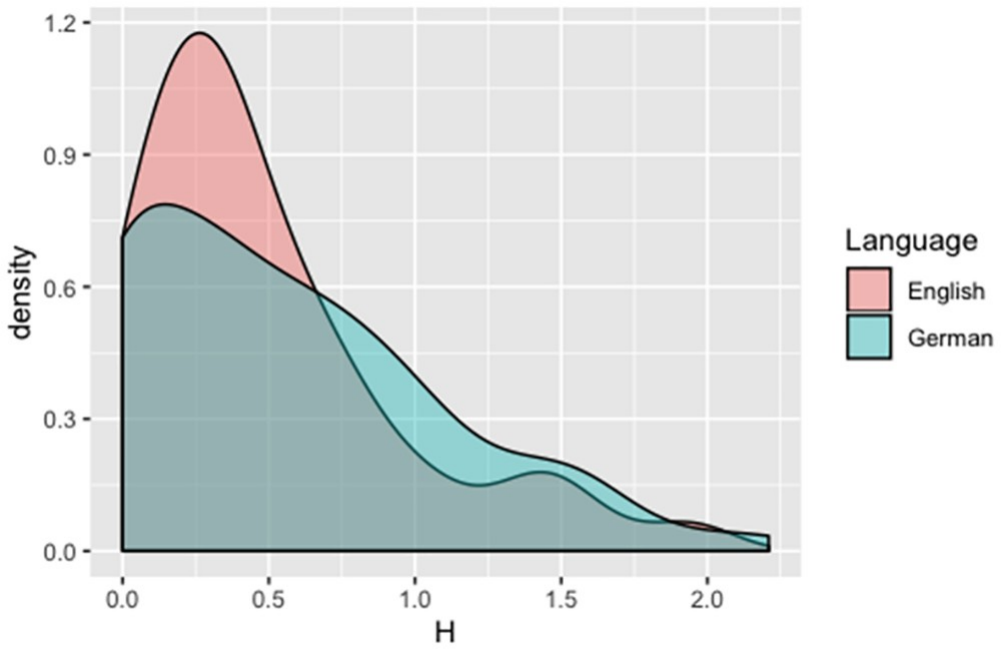
Distribution of Entropy values for German and English adults.

The third, theoretically relevant question, was whether or not the observed Entropy differed between the English and German readers. To visualize the distribution of the Entropy values, we generated a density plot of the English and German Entropy values (see Fig 1). Fig 1 shows that the distribution is right-skewed, with many items having an Entropy value close to zero. Therefore, we performed a Mann-Whitney test, with language as a predictor of Entropy. The difference in Entropy between English and German was not significant, *W* = 3710, *p* = 0.33, 95%*CI* = [−0.15, 0.10]. The pronunciation plausibility analysis confirmed the non significance of the original analysis: *W* = 3689, *p* = 0.29, 95%*CI* = [−0.15, 0.09].

Tables 2 and 3 in S1 Appendix show the participants pronunciations to the ten items with the highest Entropy values. Participants mistakenly read some pseudowords as real words, but there was no significant difference in number of real words pronunciation between German(*m* = 0.05, *sd* = 0.22) and English adults(*m* = 0.02, *sd* = 0.16): *p* = 0.18. A list can be found in the Table 1 in S1 Appendix.

Both in English and in German, we found a non-normal distribution of Entropy values, with many Entropy values being close to zero (suggesting consistent pronunciations across participants). Thus, even in the English orthography, despite a number of items which result in a high degree of variability of responses, there is often a consensus about how to pronounce a given item (see also [20] for a similar conclusion). Mousikou, Sadat, Lucas and Rastle [20] argue that this agreement in English pseudowords pronunciation, despite the inconsistency of its orthography, can be explained by the influence that a pseudoword’s orthographic neighbors have on its pronunciation (for example *key* could interfere with the pronunciation of *kuy*), and by the fact that, even if a grapheme maps into several phonemes (<i> can be read as /ai/, /ɪ/ or /ɜ:/), participants will tend to pronounce it with the phoneme that is most frequently associated with it. For example, participants read the pseudoword “dize” mostly as /daiz/ (14 participants) and less likely as /dɪze/ (5 participants).

In German, the analysis of the ten items with the highest Entropy values revealed that there were few phonotactic properties that were not systematically applied to pseudowords. For example, the final consonant devoicing phenomenon, which normally makes the voiced final consonant voiceless in words (*Rad—bike* being read as */rat/*) was not always applied: the pseudoword *gund* was read only half of the time *gunt*. Two context correspondences also triggered higher Entropy values: the first concerns the pronunciation of the grapheme <s> in front of the grapheme <p>. Normally, in words like *Sport*, the <s> would be read as /ʃ/. However, in our data, participants read pseuwords like *sprau* either /ʃprau/ or /sprau/. Similarly, the grapheme <n> before the final grapheme <g> should give the phoneme /ɳ/, but participants productions in pseudowords like *quang* varied from /ɳ/, /ɳg/ to final /n/.

The present cross-linguistic comparison did not reveal differences in Entropy between English and German. Previous studies have found differences in accuracy as a function of orthographic depth (e.g., [21]). Since a low accuracy should be evident with high Entropy, we expected to find higher Entropy values in English compared to German. However, most previous reading aloud studies were conducted with children [23–25]. Adult studies have often used lenient marking criteria, and accuracy tends to reach ceiling. Thus, there is little evidence to suggest that cross-linguistic differences in accuracy or pronunciation variability persist into adulthood. The current analysis overcomes this limitation by using Entropy instead of a lenient marking criterion and suggests that, in adulthood, orthographic depth has a minimal influence on the heterogeneity of pseudoword reading aloud responses.

## Experiment 2: Entropy in German monolingual children and German/English bilingual children

The aim of the second experiment was to test whether there were differences in Entropy in a younger population: that is, in primary school children. Although the results of Experiment 1 demonstrate that the Entropy of pseudoword reading aloud responses did not differ across German and English-speaking adults, this does not rule out that Entropy differences may exist between German and English-speaking primary school children who are still in the process of learning to read. Entropy differences in adults may be washed out by the fact that the skilled reading system has already established an optimal prediction system for letter-sound correspondences, which may not yet have developed to the same level of precision in developing readers. Experiment 2 put this hypothesis to test by acquiring data from monolingual German children and German/English bilingual children in grades 2, 3, and 4 reading matched pseudowords both in German and in English. This allowed us to compare Entropy within the same items and participants across grade (in German monolingual children) and across orthographies within the same participants.

Overall, we predicted higher Entropy in younger than in older children, because the knowledge of the GPCs may not be full developed, which could lead to a greater level of noisiness in their decision about how to pronounce a given GPC [12]. Moreover, Entropy was expected to be higher for the English than German items, because the depth of English may make it more difficult for children to learn the GPCs. Such a finding would be in line with previous studies, suggesting that pseudoword reading aloud accuracy is lower in English than in shallower orthographies (e.g., [21]). Finally, we hypothesized that Entropy may be higher in bilingual children than monolingual children, because the knowledge of GPCs within one language may interfere with the pseudowords reading aloud responses in the other language [30].

### Methods

#### Participants

Six groups of children participated in this experiment: Three groups of monolingual German children, enrolled in in grade 2 (N = 22), grade 3 (N = 19), and grade 4 (N = 22) (for a more detailed description of this sample, see [12]) were recruited in German primary schools, as well as, three groups of German/English bilingual children attending grade 2 (N = 12), grade 3 (N = 5), and grade 4 (N = 5) of a bilingual primary school in Australia. Prior to testing informed consent was obtained from children’s parents. The data reported here were not analyzed or reported in Schmalz et al (2020) study. Participants’ German proficiency of both bilingual and monolingual children was tested with the standardized reading test SLRT (Salzburg Reading and Spelling Test [41]). The median percentile for monolingual children was 50.50 (*min* = 8, *max* = 94;*sd* = 28.36) and for bilingual children 45.50 (*min* = 5, *max* = 88;*sd* = 21.87). A t-test comparing Monolingual German proficiency and Bilingual German proficiency in grade 2 (the comparison between bilingual and monolingual participants is done for grade 2 children only) revealed no significant difference between the two groups: *t* = 0.73, *p* = 0.46.

#### Materials

The same items as in Experiment 1 were used.

#### Procedure

The experimental procedure, as well as the transcription of the audio files and calculation of Entropy for each pseudoword, was identical to Experiment 1. The German monolingual children read the German pseudowords, and the bilingual children read both German and English pseudowords. The bilingual children were tested on separate days. On one day, to avoid any external, cross-linguistic influences on the children’s reading behavior, the experimenter spoke only German to them and they performed a number of additional German reading tasks, and on the other day, the experimenter spoke only English and they performed a number of additional English reading tasks (which are not reported here). The order of session was counterbalanced across participants, so half the children started with the German session and the other half of the children started with the English session.

### Results and discussion

We excluded non-responses before calculating Entropy. This resulted in a loss of 179 trials (3.16% of all trials) for the monolingual sample, and 77 trials (3.10% of all trials) for the bilingual sample. The data from this experiment were used to compare Entropy across three dimensions: language, bilingualism and age.

#### Language

**Fig 2:**
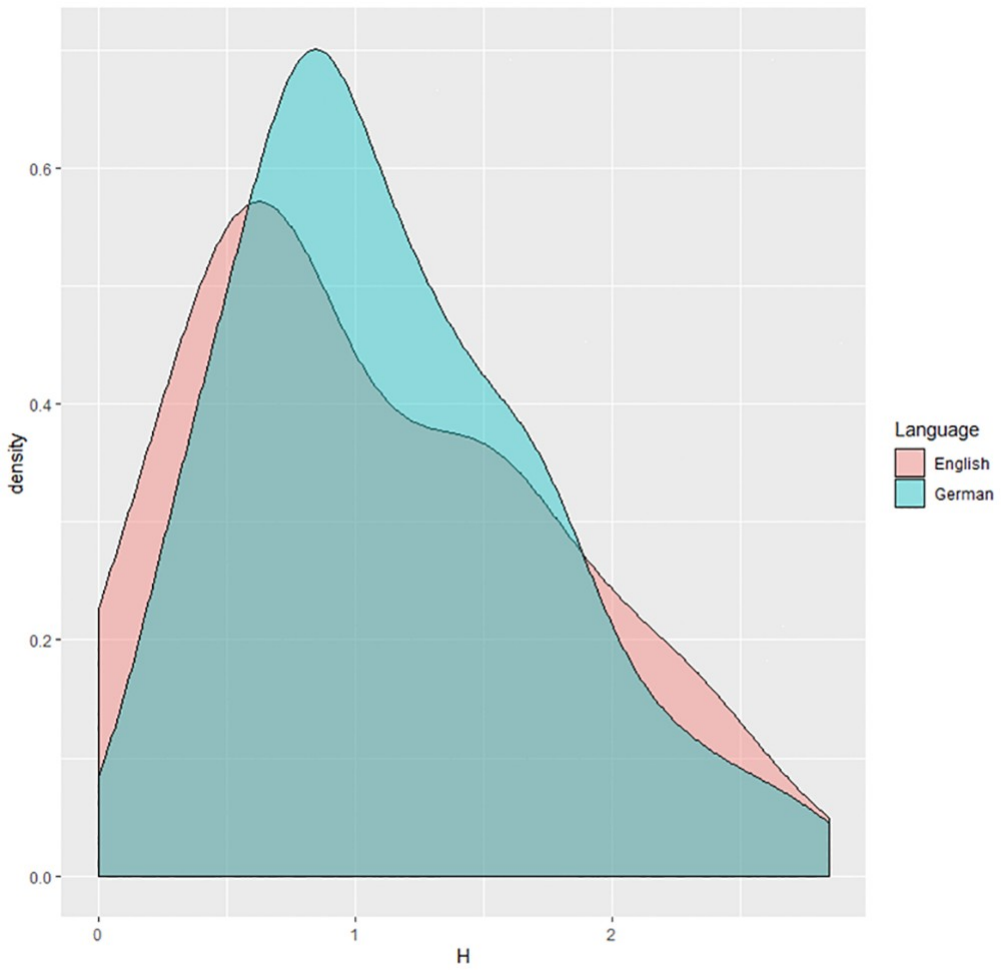
Distribution of Entropy for German/English bilingual children reading German vs English items. The dashed lines are the medians for each orthography.

Firstly, we used language as a predictor (bilingual children reading German vs. the same bilingual children reading English) and performed a Mann-Whitney test: there was no significant difference in Entropy between languages: *W* = 3660, *p* = 0.26, 95%*CI* = [−0.28, 0.10]. The pronunciation plausibility re-analysis confirmed the results from the original analysis *W* = 4238, *p* = 0.59, 95%*CI* = [−0.10, 0.26]. In German, the median of the Entropy value, across all items, was 0.99 (min = 0.27, max = 2.77), whereas in English it was 0.91 (min = 0, max = 2.83). See Fig 2 for the distribution of Entropy.

Tables 4–6 in S1 Appendix show the pronunciations of bilingual children reading the ten English-like pseudowords with the highest Entropy values, while Tables 7–9 in S1 Appendix show the same children reading the ten German-like pseudowords.

A direct observation of children’s responses to German and English items showed that both adults and children produced the same alternative pronunciations to certain units. For example, in German, both groups were not uniform regarding the final consonant devoicing phenomenon that is, on the contrary, systematically applied on words (see Tables 2, 6–12 in S1 Appendix). The pseudoword “fold” was read either /folt/ or /fold/. Similarly, when in English pseudowords the letter <r> was preceded by a vowel, both adults and children were divided whether to read it or not. Note that the participants were native Australian speakers: in Australian English, for monosyllabic words, vowels followed by the letter *r* always form a multi-letter rule (but not in multisyllabic words:“kangaroo”is read, for example, /kægəru:/). Tables 3, 5–7 in S1 Appendix show such similar instances. As for the number of lexicalization errors, participants did not significantly read German items as real words (*m* = 0.035, *sd* = 0.89) more than English items (*m* = 0.038, *sd* = 0.19): *p*-*value* = 0.74.

Furthermore, we calculated a correlation matrix for bilingual children reading English items in grade 2, and for bilingual children reading German items in grade 2, similarly to Experiment 1.

For bilingual children reading English items, we calculated the agreement between the scorers using Cohen’s Kappa. In this case scorers were in a strong agreement (*k* = 0.70). Entropy correlated negatively with both accuracy scoring (s2, *r* = −0.62, *p* < 0.001, s1, *r* = −0.61, *p* < 0.001). In fact, higher accuracy means lower Entropy. Naturally we also found significant correlations between Entropy and number of pronunciations (*r* = 0.96, *p* < 0.001) and Entropy and percentage of the most common response (*r* = −0.96, *p* < 0.001). As for the German items, Entropy correlated negatively with accuracy scoring (s1, *r* = −0.75, *p* < 0.001) and with the percentage of the most common response (*r* = −0.93, *p* < 0.001), while positively correlating with number of different pronunciations (*r* = 0.94, *p* < 0.001). Tables 4 and 5 show the correlation matrix.

**Table 4:** Intercorrelations for bilingual children reading German items (Exp 2).

Measure	1	2	3	4
1. Entropy	-	-.75*	.94*	-.93*
2. acc	-.75*	-	-.73*	.64*
3. n_asw	.94*	-.73*	-	-.76*
4. perc	-.93*	.64*	-.76*	-
n = 90				

Note: n_asw = number of different pronunciations per pseudowords, acc = scored accuracy, perc = percentage of the most common response,

* = significant result.

**Table 5:** Intercorrelations for bilingual children reading English items (Exp 2).

Measure	1	2	3	4	5
1. Entropy	-	-.62*	-.61*	.96*	-.96*
2. acc_s2	-.62*	-	.51*	-.59*	.64*
3. acc_s1	-.61*	.51*	-	-.60*	.53*
4. n_asw	.96*	-.59*	-.60*	-	-.89*
5. perc	-.96*	.64*	.53*	-.89*	-
n = 90					

Note: n_asw = number of different pronunciations per pseudowords, acc_s1 and acc_s2 = accuracy scored by scorer 1 and 2, perc = percentage of the most common response,

* = significant result.

#### Bilingualism

**Fig 3:**
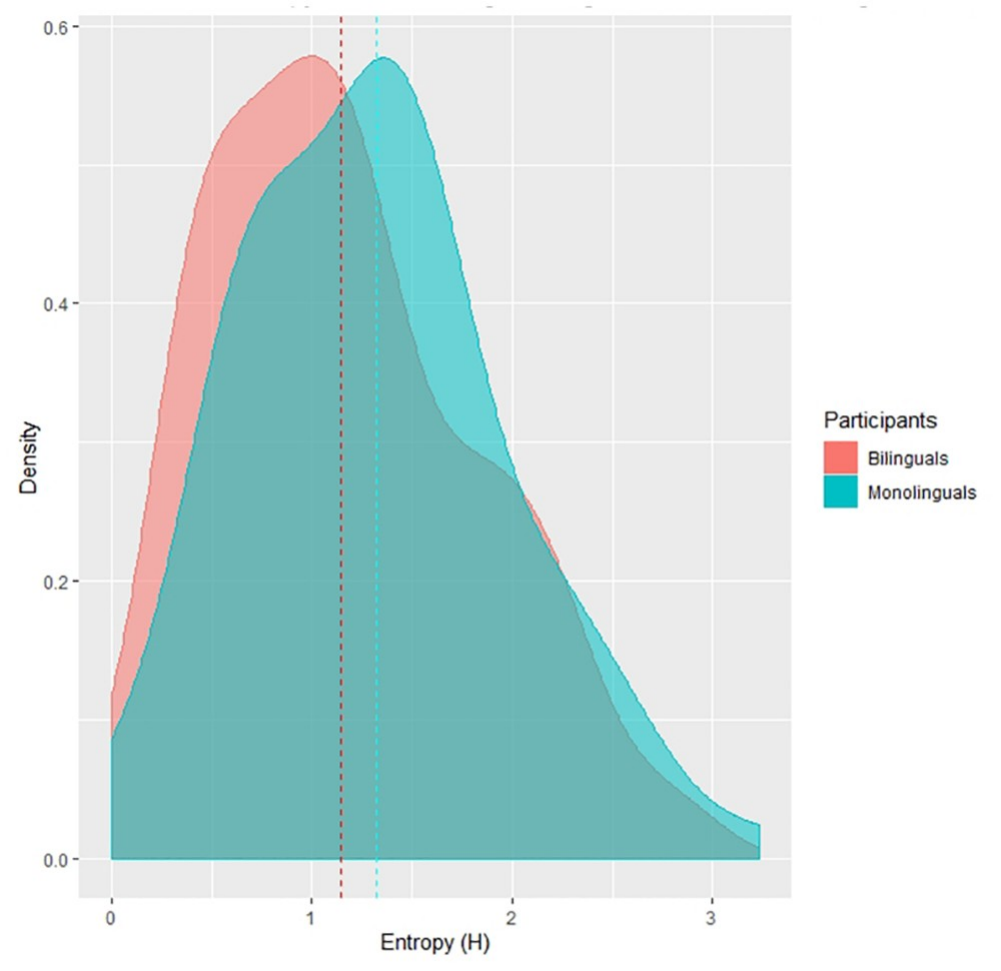
Distribution of Entropy for German/English bilingual and German monolingual children. The dashed lines are the medians for each orthography.

Secondly, we investigated whether bilingualism affected Entropy (German monolingual vs German/English bilingual children reading the same items in German). Only participants from grade 2 were included in this comparison, since the number of participants from those groups was rather similar (N = 22 monolingual, N = 13 bilingual). The median of the Entropy value for the bilingual group was 1.14 (min = 0, max = 2.81), while it was 1.32 (min = 0, max = 3.22) for the monolingual group (see Fig 2). We also performed a Mann-Whitney test to see whether there was a difference in Entropy values for the second contrast, but again we found no significant difference: *W* = 4144, *p* = 0.79, 95%*CI* = [−0.10, 0.27]. The pronunciation plausibility analysis confirmed the marginal significance of the result *W* = 3452, *p* = 0.08, 95%*CI* = [−0.36, 0.26]. This result indicates that, although there was a marginally significant difference between the two groups (p < 0.1), bilingualism did not increase answer variability (see Fig 3). This is in line with studies which show that learning an orthography that is more transparent than the other, if anything, improves the understanding of the deeper orthography GPCs [42, 43].

The current result diverges to some degree from Treiman, Kessler and Evans [30] who found that exposure to a second language affects graphemes-phonemes correspondences of the first language. In this study, native English speakers learning French applied French fronting context rule while reading<c>and<g>graphemes in English word and pseudowords. Those students took into account the following vowel to determine pronunciation, and so much more than students who were not studying a second language. Translated to Entropy, pronunciation variability in students learning a second language was lower compared to students who did not undertake a second (romance) language class. The difference with our results might be due to the fact that our participants were bilingual English-German (two Germanic languages) children (and not university students), and we could only find limited occurrences of GPC interference from English to German (for example, the German item “loo” was read /lu/ instead of /lo:/). Therefore the findings of the present study and those from Treiman, Kessler & Evans are not in direct contradiction, given the nature of participants (bilingual pupils being proficient in two languages, compared to monolingual English-speaking students, who just started to learn French as a second language) and nature of direction (interference between equally mastered languages, compared to interferences from L2 to L1) even though our result was only marginally significant. In the real word data, there was no significant difference in real words reading between bilinguals (*m* = 0.035, *sd* = 0.18) and monolingual German children reading in German(*m* = 0.42, *sd* = 0.19): *p*-*value* = 0.40. A list can be found in Table 13 in S1 Appendix.

#### Grade

**Fig 4:**
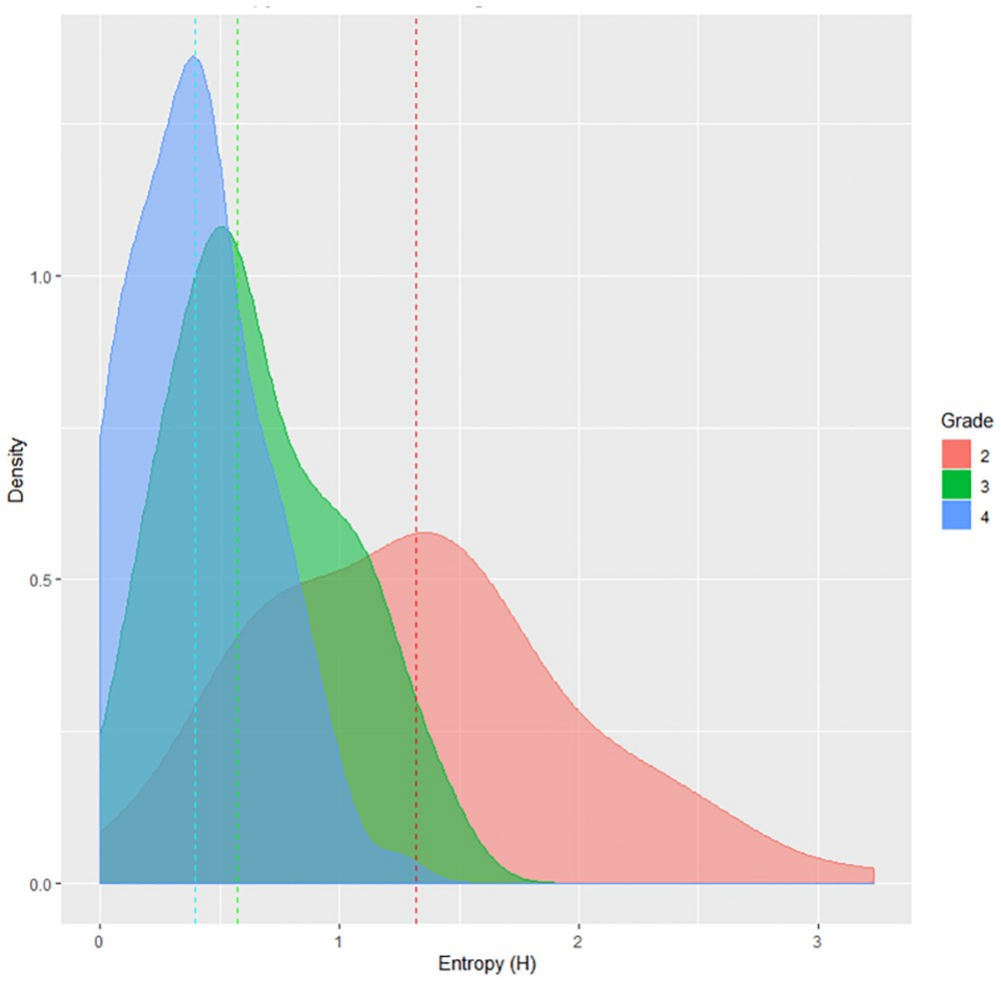
Distribution of Entropy for German monolingual children across grades. The dashed lines are the medians for each orthography.

Finally we used grade as a predictor of Entropy (we compared German monolingual children from grade 2, 3 and 4 across grades). We performed a Mann-Whitney test (between grades 2 and 3; 2 and 4; 3 and 4) and calculated Entropy medians. In grade 2 the median of Entropy values was of 1.31 (min = 0; max = 3.22), 0.58 in grade 3 (min = 0; max = 1.51) and 0.39 in grade 4 (min = 0; max = 1.25) (Fig 4). The Mann-Whitney test showed significant differences between grade 2 and 3: *p* < 0.001, 95%*CI* = [0.45, 0.79]; grade 2 and 4: *p* < 0.001, 95%*CI* = [0.72, 1.23] and grade 3 and 4: *p* < 0.001, 95%*CI* = [0.14, 0.35]. The pronunciation plausibility analysis confirmed the significance of all comparisons: grade 2 and 3: *p* < 0.001, 95%*CI* = [−0.57, −0.30]; grade 2 and 4: *p* < 0.001, 95%*CI* = [−0.79, −0.54] and grade 3 and 4: *p* < 0.001, 95%*CI* = [0.14, 0.31]. As we can see from the Entropy values medians changing across grades (see Fig 4), by developing and practicing their reading skills children gradually became more acquainted with the GPCs of their language, and their answer variability decreased. This result is in line with the findings of [12], who found decreasing Entropy in vowel pronunciation variability (but not in consonant pronunciation variability, which was not investigated) as a function of grade. In real words, there was no significant difference in lexicalizations between grades(grade 2: *m* = 0.41, *sd* = 0.19; grade 3: *m* = 0.04, *sd* = 0.21; grade 4: *m* = 0.03, *sd* = 0.17): *p* = 0.594 for the comparison between grade 2 and grade 3; *p* = 0.524 between grade 3 and grade 4; and *p* = 0.274 between grade 2 and grade 4).

We calculated correlations between Entropy and other measures for all grades (Tables 6–8). In grade 2 Entropy correlated significantly with accuracy (*r* = −0.71, *p* < 0.001), the number of different pronunciations (*r* = 0.92, *p* < 0.001) and the percentage of the most common response (*r* = −0.91, *p* < 0.001). In grade 3, correlations remained significant for the number of different pronunciations (*r* = 0.90, *p* < 0.001) and for the percentage of the most common response (*r* = −0.93, *p* < 0.001), but the correlation with accuracy was not significant (*r* = −0.05, *p* = 0.62). The same scenario from grade 2 repeated in grade 4: Entropy significantly correlated with accuracy (*r* = −0.70, *p* < 0.001), the number of different pronunciations (*r* = 0.91, *p* < 0.001) and the percentage to the most common response (*r* = −0.93, *p* < 0.001).

**Table 6:** Monolingual German children in grade 2 (exp 2).

Measure	1	2	3	4
1. Entropy	-	-.71*	.91*	-.91*
2. acc	-.71*	-	-.70*	.58*
3. n_sw	.91*	-.70	-	-.73*
4. perc	-.91*	.58	-.73*	-
n = 90				

Note: n_asw = number of different pronunciations per pseudowords, acc = scored accuracy, perc = percentage of the most common response,

* = significant result.

**Table 7:** Monolingual German children in grade 3 (exp 2).

Measure	1	2	3	4
1. Entropy	-	-.05	.90*	-.93*
2. acc	-.05	-	-.13	-.03
3. n_asw	.90*	-.13	-	-.72*
4. perc	-.93*	-.03	-.72*	-
n = 90				

Note: n_asw = number of different pronunciations per pseudowords, acc = scored accuracy, perc = percentage of the most common response,

* = significant result.

**Table 8:** Monolingual German children in grade 4 (exp 2).

Measure	1	2	3	4
1. Entropy	-	-.70*	.91*	-.93*
2. acc	-.70*	-	-.79*	.53*
3. n_asw	.91*	-.79*	-	-.71*
4. perc	-.93*	.53*	-.71*	-
n = 90				

Note: n_asw = number of different pronunciations per pseudowords, acc = scored accuracy, perc = percentage of the most common response,

* = significant result.

The pronunciations of the ten items with the highest Entropy values are listed in Tables 10–12 in S1 Appendix, andfor a list of pseudowords read as realwords are listed in Table 14 in S1 Appendix (grade 2: *m* = 0.04, *sd* = 0.17; grade 3: *m* = 0.04, *sd* = 0.21; grade 4: *m* = 0.03, *sd* = 0.17).

## Experiment 3: Entropy in French and Italian children

The cross-linguistic contrast in Experiments 1 and 2 relied on a comparison of German and English pseudowords and did not reveal any cross-linguistic differences in English and German speaking adults and children.

However, since we used a bilingual sample to search for cross-linguistics differences in children, it may be the case that the knowledge of one shallow orthography (German) had a facilitatory effect on the knowledge of the deeper language (English). One possible explanation is that the children’s knowledge of two different orthographies enhanced their understanding of GPCs. Many studies on bilingualism, in fact, suggest that bilingual children possess greater metalinguistic awareness [30, 44–46], defined as “the explicit knowledge of the structural components of their orthography” [43].

At the same time, we wanted to test whether complexity, rather than unpredictability affected Entropy. Since English orthography is considered both unpredictable and complex, and could not serve for this purpose, we chose to collect data from two more groups of children, French and Italian fourth graders. By comparing them, we were able to also assess the effect of complexity on Entropy: French, compared to other European orthographies, has many complex correspondences, while Italian has relatively few, with unpredictability being relatively low in both orthographies [28].

### Methods

#### Participants

A group of Italian fourth graders (n = 33) and a group of French fourth graders (n = 29) were recruited for this experiment. Children’s parents agreed to the participation by signing an informed consent.

#### Materials

The children read aloud a list of 40 pseudowords, generated from a list of cognate words (with similar orthograpny and the same meaning in both languages, like “maternité” and “maternità”- maternity). Pseudowords were matched in number of syllables, number of letters, orthographic neighborhood entity and base-word frequency.

#### Procedure

First, during a preliminary phase, we ensured that no children had learning disorders. One French child who was already diagnosed with dyslexia was excluded. Second, we administered the pseudoword reading aloud task to each participant. The procedure was identical to Experiments 1 and 2.

### Results and discussion

**Fig 5:**
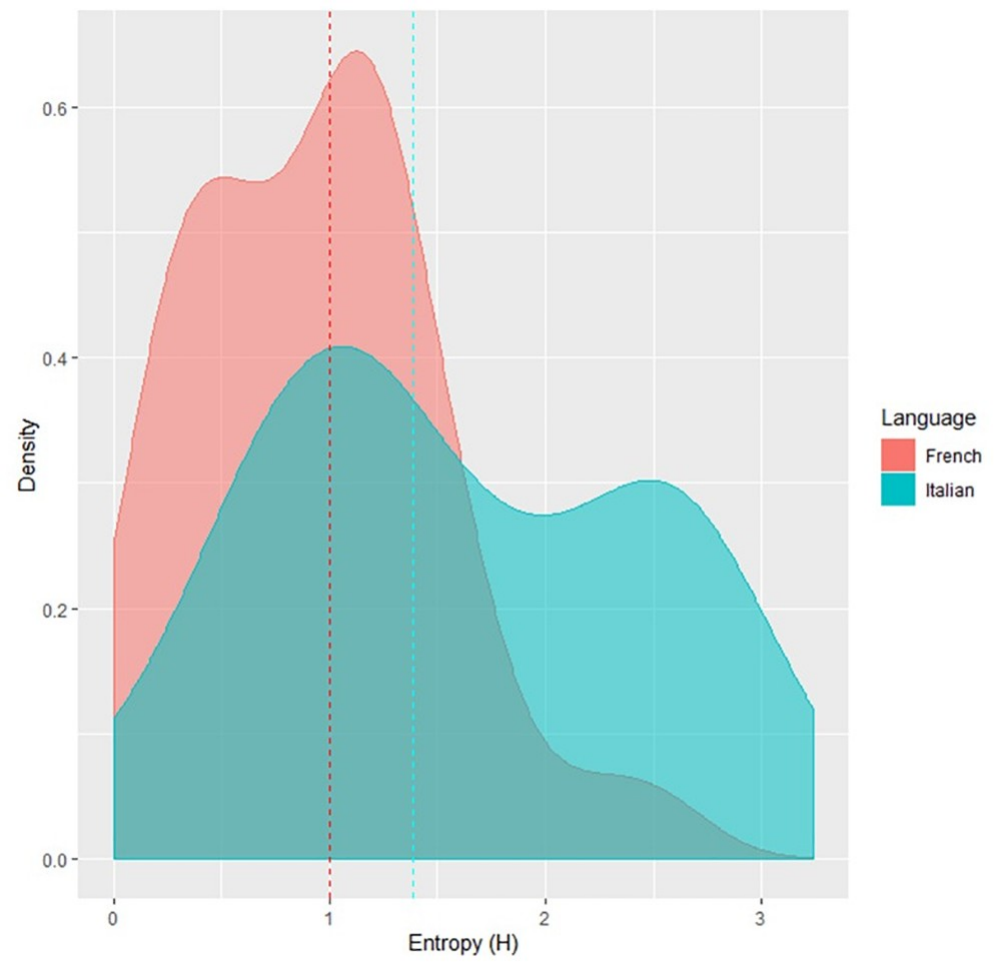
Distribution of Entropy values in French and Italian children. The dashed lines are the medians for each orthography.

Entropy was calculated using the same script and formula of the other experiments. For French speaking children the median of the Entropy value was 0.99 (min = 0, max = 2.53), while for Italian speaking children it was 1.38 (min = 0, max = 3.24). We then performed a Mann-Whitney test between French and Italian items, which showed a significant effect *W* = 460, *p* < 0.05, 95%*CI* = [−0.98, 0.22], reflecting higher Entropy in Italian than French children (see Fig 5). Once again, the pronunciation plausibility analysis confirmed this result: *W* = 468.5, *p* < 0.05, 95%*CI* = [−0.81, 0.19].

Cohen’s kappa calculation revealed that scorers were in a moderate agreement for French data (*k* = 0.57) and in a nearly perfect agreement for Italian data (*k* = 0.92). The fact that for Italian data the scorers were in a nearly perfect agreement does not come as a surprise: since it is a shallow orthography, and has an almost perfect isometric mapping between graphemes and phonemes, it is easier and more straightforward to determine which pronunciation can be considered correct or wrong.

In the French data we found a significant, negative correlations between accuracy and Entropy (scorer 1 *r* = −0.49, scorer 2 *r* = −0.58): *p* < 0.001. Again, this was expected, as higher accuracy implies lower Entropy. A significant, positive correlation was found with number of pronunciations (*r* = 0.75, *p* < 0.001) and a negative correlation was found with the percentage of the most common response (*r* = −0.81.*p* < 0.001). In Italian, Entropy was significantly correlated with number of different pronunciations (*r* = 0.94, *p* < 0.001) and percentage of most common response (*r* = −0.93, *p* < 0.001), but surprisingly not with the accuracy judgements (s1 *r* = 0.08, *p* = 0.63, s2 *r* = 0, *p* = 0.99).

Tables 9 and 10 show the correlation matrix.

**Table 9:** Intercorrelations for French children (Exp 3).

Measure	1	2	3	4	5
1. Entropy	-	-.58*	-.49*	.75*	-.81*
2. acc_s2	-.58*	-	.74*	-.48*	.71*
3. acc_s1	-.49*	.74*	-	-.51*	.62*
4. n_asw	.75*	-.48*	-.51*	-	-.63*
5. perc	-.81*	.71*	.62*	-.63*	-
n = 40					

Note: n_asw = number of different pronunciations per pseudowords, acc_s1 and acc_s2 = accuracy scored by scorer 1 and 2, perc = percentage of the most common response,

* = significant result.

**Table 10:** Intercorrelations for Italian children (Exp 3).

Measure	1	2	3	4	5
1. Entropy	-	.00	.08	.94*	-.93*
2. acc_s2	.00	-	.97*	.05	-.01
3. acc_s1	.08	.97*	-	.15	-.08
4. n_asw	.94*	.05	.15	-	-.79*
5. perc	-.93*	-.01	-.08	-.79*	-
n = 40					

Note: n_asw = number of different pronunciations per pseudowords, acc_s1 and acc_s2 = accuracy scored by scorer 1 and 2, perc = percentage of the most common response,

* = significant result.

We then analyzed the responses to the ten items with the highest Entropy values, in order to qualitatively assess which factors may lead to higher Entropy value (see Tables 16 and 17 in S1 Appendix). A comparison between Italian and French children revealed that Italian participants misread items as real words more often than French participants (p < 0.05). A list of real words readings can be found in Table 15 in S1 Appendix.

The qualitative analysis performed on French children’s answers showed that alternative answers were given especially when pseudowords contained inconsistent graphemes (as the sibilant <s>) or nasal sounds (e.g. <am>, <en>, <aim>). As for the <r> grapheme, it has been previously shown that its corresponding phoneme /ʁ/ is challenging for children to acquire, and its acquisition occurs very late [47]. Our results suggest that grade 3 children’s GPCs are not fully developed yet. Another element that created alternative readings was the pronunciation of final consonants that are not normally read in real words, such as <t>, <r>, <d> and <s> in pseudowords as *stort, fratis, buffat, antobus, gord and cosputer*. Our participants were very divided regarding this issue, and since we got the same alternative answers from French adults (Exp. 4), we concluded that age reading skills are not possible causes of these answers. These results are consistent with [48] who found that French-speaking participants pronounced letters in nonwords that are typically silent in words.

Italian children’s responses were affected by the pseudowords’ orthographic neighbors or by the recognition of the base word itself; this is the case for the grapheme <g> read as the phoneme /ʒ/ for the pseudowords “benge” (baseword: “beige”) and “darage” (baseword: “garage”). Children who produced this phoneme (which is not in the Italian phoneme inventory) recognised the French loanwords and had knowledge of their irregular reading. In regard to the other occurrences, Italian children did not apply phonotactic cues that normally indicate which phoneme must be pronounced. For example, when <s> and <z> are surrounded by vowels, their voiced alternative (/z/ and /dz/, respectively) should be produced. Therefore, <anisale> should be read as /anizale/ and <vazionalità> as /vadzionalita/. This voicing assimilation phenomenon, which is the norm in the central and northern areas of Italy, is however not common in the southern regions of Italy, and specifically not in Sardinia (the native region of our participants). Moreover, these phonemes are often considered allophones by Italian speakers, depending on their geographical origin. Consequently,the alternative pronunciations of some inconsistent graphemes are not considered wrong or not fitting, and individual responses can vary even within the same participant (who will produce the alternative readings of that grapheme in a non-systematic fashion).

Against our predictions, the median Entropy value was lower in French than Italian children, suggesting that complexity may not increase pseudoword reading aloud Entropy. One explanation for the higher Entropy values in Italian could lie in the characteristics of the items themselves. In order to create a set of cognate items, we chose similar words in Italian and French, and then generate pseudowords by changing letters. Since Italian syllabic structure is simpler than in French [21], it is possible that French children had to read items that were not representative of their structural complexity. For example, “pizza”, a common word in both languages that reflects the Italian syllabic structure [CVCV], has a simpler syllabic structure than “fauteuil” [CVCVC], a typical French word, and also fewer diphtongs and inconsistent graphemes). The goal of Experiment 4 was to rule out this possibility by providing sets of items that are truly representative of the participants’ languages.

## Experiment 4: Entropy in English, French, and German adults reading non-matched pseudowords

The results of Experiments 1-3 did not reveal any cross-linguistic differences that would have suggested that deeper orthographies lead to greater variability in pseudoword pronunciations. However, in these experiments, items were strictly matched on various psycholinguistic properties across orthographies (syllable structure, number of letters, frequency, orthographic neighborhood and so forth). The advantage of this setup is that researchers can control for a number of psycholinguistic properties that can influence participants’ reading behavior. However, one disadvantage is that cognate pseudowords may not always be representative of the types of words that the readers typically encounter in their native orthography, and therefore had the potential to reduce variability in participants’ responses across languages.

This considered, in the last experiment, we created two different sets of items. For the first set, we created pseudowords that only matched on frequency, and not, for example, on syllable structure or number of letters. We based this design on the idea of a frequency-matched reading aloud study [49]. In this study Ellis et al. argued that matching items on all possible characteristics creates item sets which are unnatural for most of the orthographies. Note that this problem persists in the cognate design: for example, the German/English cognate “Zeitgeist”, the spelling is typical, regular and predictable in German, but strange and irregular in English. The reverse is true for the cognate “steak”. The solution proposed by Ellis et al. [49] was to allow words to vary across languages on all dimensions except frequency. All words from a corpus are divided into frequency bands, and an equal amount of words is randomly chosen from each frequency band from each language. Word frequency is a measure of the frequency with which participants are expected to have encountered this word. Thus, if frequency is matched across languages, participants’ familiarity with a given word is kept constant. All other item-level characteristics vary, but this variation is systematic, as it reflects the orthographies’ characteristics. In the current study, we were interested in pseudoword rather than word reading. Therefore, we first chose a series of words, using the frequency-matched design, and then created a set of pseudowords from these words using the same procedure across orthographies. The advantage of this approach is that pseudowords will inherit properties that are characteristic of the orthography, such as length and bigram frequency.

In the second set of items, we took the opposite approach. We created pseudowords which were identical across orthographies, and which were equally untypical of real words in all orthographies in question (orthographic neighborhood of 0). These were pseudwords with a CVCVCV structure, containing only letters which occur frequently in all three orthographies in question.

In Experiments 1-2, furthermore, the items were all monosyllabic. In Experiment 4, we relaxed this constraint. In general, pronunciations become less consistent when polysyllabic words are considered [51]. Therefore, the presence of polysyllabic pseudowords gives more scope for readers of deep orthographies to provide variable pronunciations.

### Methods

#### Participants

Participants were 16 students from universities in southern Germany, 28 students from a university in southern France, and 39 students from a university in Australia. They participated in exchange for course credit or payment.

#### Materials

As outlined in the previous section, we chose two subsets of items. For the first subset, we selected a number of words from each language using a frequency-matched design, following the same procedure as [49]. We randomly selected words from different frequency bands: 10 words each with a log-frequency between 0 and 0.5, between 0.5 and 1, between 1 and 1.5, and between 1.5 and 2 [51–53]. We then created pseudowords for each item in each language, using the software Wuggy [54]. Wuggy’s algorithm generates pseudowords that are similar to the input words in terms of subsyllabic structure, bigram frequency, and orthographic neighborhood. Thus, we obtained 40 pseudowords, based on real words varying in frequency, for each orthography.

In the second set, the items were equally easy to pronounce, we assembled 20 pseudowords from simple CV syllables. Each pseudoword had three syllables, and an orthographic neighborhood of zero. Thus, in all languages, items were equally dissimilar to real words.

#### Procedure

The two sets of pseudowords were presented to the participants in a mixed random order, using the software DMDX [55]. The participants saw each item on the screen for 2.5 seconds or until the voice key was triggered, and were instructed to read aloud the items as fast as possible, while being as accurate as they could be. The data was then transcribed, for each orthography, by a native speaker.

### Results and discussion

**Fig 6:**
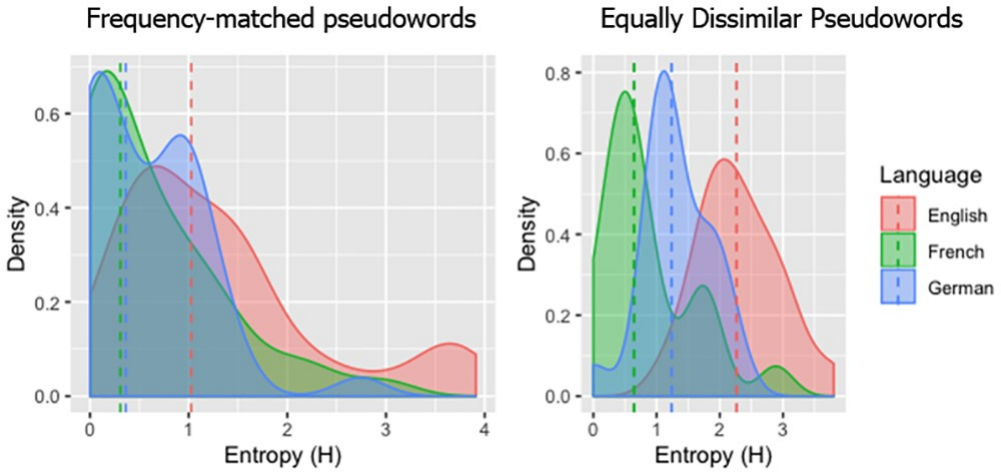
Distribution of Entropy values for French, German and English adults. The dashed lines are the medians for each orthography.

We excluded all non-responses (12 trials for English, no trials for French, and 3 trials for German, <1% of the data) before further processing. Entropy was calculated, for each language separately, as in the previous experiments (see Fig 6).

Since Fig 6 showed a non-normal Entropy distribution was compared across languages in a pairwise manner, we used the Mann-Whitney test.

#### Word-like pseudowords

First, in the comparison of the word-like pseudowords, which were derived from the frequency-matched words, the median Entropy was 1.03 for English (min = 0, max = 3.91), 0.31 for French (min = 0, max = 2.98), and 0.36 for German (min = 0, max = 2.74). The Mann-Whitney tests showed a significant difference between English and French, *W* = 1160, *p* < 0.001, 95%*CI* = [0.30, 0.88], between English and German, *W* = 1177, *p* < 0.001, 95%*CI* = [0.22, 0.83], but no significant difference between French and German, *W* = 800, *p* > 0.9, 95%*CI*[−0.24, 0.23]. Again the results were confirmed by the pronunciation plausibility analysis: comparisons between English and French (*W* = 1161.5, *p* < 0.001, 95%*CI* = [0.32, 0.73]) and English and German remained significant (*W* = 1089, *p* = 0.005, 95%*CI* = [0.16, 0.64]), while the non-significance of French and German comparison was corroborated (*W* = 705, *p* = 0.34, 95%*CI* = [−3.53, 8.76]).

Three multiple linear regressions were calculated including language, length, number of syllables, baseword and bigram frequency, orthographic neighborhood, phonological neighborhood and BodyN. Baseword frequency and Body Neighborhood were calculated using Leipzig Corpora Collection [56–58], while Bigram frequency, orthographic and phonological neighborhood were calculated thanks to the Clearpond database [59].

Results indicated that none of these variables, apart from Language (in the comparison between English and French, and English and German) and Length (in the comparison between English and Grench) were significant predictors in the model (see Tables 11–13), which confirmed the results of the Mann-Whitney tests.

**Table 11:** Multiple regression, English-French, word-like pseudoword.

	Model 1		Model 2	
(Intercept)	0.39, (0.15)*		0.03, (0.42)	
Language	−0.77, (0.21)***		−0.79, (0.24)**	
Length			0.28, (0.13)*	
Syllables count			0.22, (0.25)	
Baseword Frequency			0.07, (0.11)	
Orthographic N			−0.00, (0.10)	
Phononological N			−0.08, (0.11)	
Body N			−0.15, (0.13)	
Bigram Frequency			−0.08, (0.10)	
R^2^	0.15		0.30	
Adj. R^2^	0.14		0.22	
Num. obs.	80		80	
	Estimate	Std. Error	t value	Pr(> |t|)
(Intercept)	0.0345	0.4183	0.08	0.9346
Language	-0.7878	0.2395	-3.29	0.0016**
Length	0.2813	0.1322	2.13	0.0368*
Syllables count	0.2212	0.2530	0.87	0.3849
Baseword Frequency	0.0687	0.1103	0.62	0.5352
Orthographic N	-0.0008	0.1025	-0.01	0.9937
Phononological N	-0.0800	0.1083	-0.74	0.4627
Body N	-0.1469	0.1291	-1.14	0.2589
Bigram Frequency	-0.0785	0.1048	-0.75	0.4562

***p < 0.001;

**p < 0.01;

*p < 0.05

**Table 12:** Multiple regression, English-German, word-like pseudoword.

	Model 1		Model 2	
(Intercept)	−0.40(0.15)**		−0.86(0.40)*	
Language	0.79(0.21)***		0.73(0.23)**	
Length			0.18(0.14)	
Syllables count			0.34(0.26)	
Baseword Frequency			−0.01(0.11)	
Orthographic N			−0.04(0.11)	
Phonological N			0.03(0.12)	
Body N			−0.16(0.12)	
Bigram Frequency			−0.10(0.11)	
R^2^	0.16		0.26	
Adj. R^2^	0.15		0.17	
Num. obs.	80		80	
	Estimate	Std. Error	t value	Pr(>|t|)
(Intercept)	-0.8563	0.4048	-2.12	0.0379*
Language	0.7289	0.2316	3.15	0.0024**
Length	0.1756	0.1373	1.28	0.2051
Syllables count	0.3392	0.2578	1.32	0.1924
Frequency	-0.0116	0.1133	-0.10	0.9189
Orthographic N	-0.0414	0.1051	-0.39	0.6947
Phonological N	0.0319	0.1219	0.26	0.7943
Body N	-0.1575	0.1236	-1.27	0.2068
Bigram Frequency	-0.0993	0.1053	-0.94	0.3489

***p < 0.001;

**p < 0.01;

*p < 0.05

**Table 13:** Multiple regression, French-German, word-like pseudowords.

	Model 1		Model 2	
(Intercept)	0.01(0.16)		−0.11(0.47)	
Language	−0.01(0.23)		−0.08(0.28)	
Length			−0.04(0.15)	
Syllables count			0.09(0.29)	
Baseword Frequency			−0.01(0.13)	
Orthographic N			−0.07(0.12)	
Phonological N			−0.09(0.13)	
Body N			−0.24(0.14)	
Bigram Frequency			−0.04(0.14)	
R^2^	0.00		0.07	
Adj. R^2^	−0.01		−0.03	
Num. obs.	80		80	
	Estimate	Std. Error	t value	Pr(>|t|)
(Intercept)	-0.1128	0.4660	-0.24	0.8093
Language	-0.0764	0.2780	-0.28	0.7841
Length	-0.0421	0.1541	-0.27	0.7856
Syllables count	0.0916	0.2885	0.32	0.7519
Baseword Frequency	-0.0069	0.1280	-0.05	0.9571
Orthographic N	-0.0665	0.1183	-0.56	0.5758
Phonological N	-0.0949	0.1306	-0.73	0.4698
Body N	-0.2436	0.1444	-1.69	0.0960
Bigram Frequency	-0.0442	0.1386	-0.32	0.7507

***p < 0.001;

**p < 0.01;

*p < 0.05

Since German and French have predictable mappings between graphemes and phonemes and are easy orthographies to read, this result is in line with our expectations. In contrast, English orthography is both complex and unpredictable, a characteristic that led to higher variability in responses (compared to orthographies which are complex, but predictable).

We then calculated agreement between English scorers. Cohen’s kappa measure revealed a moderate agreement (*k* = 0.54). Entropy correlated significantly with scorer 1’s accuracy judgment (s1 *r* = −0.44, *p* < 0.05), but not with scorer 2’s (*r* = −0.21, *p* = 0.19), with number of different pronunciations (*r* = 0.95, *p* < 0.001) and percentage of the most common response (*r* = −0.97, *p* < 0.001) As for the French data, the Cohen’s kappa for our scorers was *k* = 0.77, revealing a strong agreement. Entropy correlated significantly with both accuracy judgements (s1 and s2 *r* = −0.75, *p* < 0.001), number of different pronunciations (*r* = 0.92, *p* < 0.001) and percentage of the most common response (*r* = −0.97, *p* < 0.001). Similarly, in German data Entropy correlated with accuracy (*r* = −0.58, *p* < 0.001), number of different answers (*r* = −0.77, *p* < 0.001) and percentage of the most common response (*r* = −0.92, *p* < 0.001). Tables 14–16 show the correlation matrix.

**Table 14:** Intercorrelations for English-speaking adults reading word-like pseudowords (Exp 4).

Measure	1	2	3	4	5
1. Entropy	-	-.21	-.44*	.95*	-.97*
2. acc_s2	-.21	-	.44*	-.21	.19
3. acc_s1	-.44*	.44*	-	-.37*	.45*
4. n_asw	.95*	-.21	-.37*	-	-.86*
5. perc	-.97*	.19	.45*	-.86*	-
n = 40					

Note: n_asw = number of different pronunciations per pseudowords, acc_s1 and acc_s2 = accuracy scored by scorer 1 and 2, perc = percentage of the most common response,

* = significant result.

**Table 15:** Intercorrelations for French adults reading word-like pseudowords (Exp 4).

Measure	1	2	3	4	5
1. Entropy	-	-.75*	-.75*	.92*	-.97*
2. acc_s2	-.75*	-	.75*	-.62*	.80*
3. acc_s1	-.75*	.75*	-	-.61*	.82*
4. n_asw	.92*	-.62*	-.61*	-	-.86*
5. perc	-.97*	.80*	.82*	-.86*	-
n = 40					

Note: n_asw = number of different pronunciations per pseudowords, acc_s1 and acc_s2 = accuracy scored by scorer 1 and 2, perc = percentage of the most common response,

* = significant result.

**Table 16:** Intercorrelations for German adults reading word-like pseudowords (Exp 4).

Measure	1	2	3	4
1. Entropy	-	-.58*	.77*	-.92*
2. acc	-.58*	-	-.81*	.38*
3. n_asw	.77*	-.81*	-	-.58*
4. perc	-.92*	.38*	-.58*	-
n = 40				

Note: n_asw = number of different pronunciations per pseudowords, acc = scored accuracy, perc = percentage of the most common response, * = significant result,

* = significant result.

#### Dissimilar pseudowords

For the equally dissimilar pseudowords, the median Entropy values were 2.27 (min = 1.18, max = 3.80) for English, 0.64 for French (min = 0, max = 2.89), and 1.23 (min = 0, max = 2.29) for German. The Mann-Whitney tests showed a significant difference between English and French, *W* = 368, *p* < 0.001, 95%*CI*[1.11, 1.92], between English and German, *W* = 356, *p* < 0.001, 95%*CI*[0.58, 1.34], and between French and German, *W* = 102, *p* = 0.008, 95%*CI*[−0.91, −0.21] (the pronunciation plausibility analysis confirmed the significance of all comparisons. Between English and French: *W* = 324, *p* < 0.001, 95%*CI*[0.57, 1.43], English and German: *W* = 301.5, *p* = 0.006, 95%*CI*[0.22, 0.88] and French and German: *W* = 115, *p* = 0.02, 95%*CI*[−0.83, −0.05]). These findings indicate that reading aloud Entropy was higher in English than in either French or German, and higher in German compared to French, which is in contrast with the results of the previous experiment (please refer to the General Discussion, where we discuss this point in more detail).

As in our previous experiments, the observation of the participants’ responses to pseudowords confirmed that high Entropy values were associated with those items that contained non-consistent graphemes as <s> or <z>, context or position correspondences (like terminal devoicing in German or silent final consonants in French), different vowel lengths (especially in German) and different kind of phoneme manipulations (especially in English, like syllable manipulations [zulumu -> zumulu]). These phenomena can be seen in Tables 19–24 in S1 Appendix.

We then performed Cohen’s kappa between our scorers and correlations matrix. In English data, scorers were in a strong agreement (*k* = 0.74). Entropy correlated significantly with number of different pronunciations (*r* = 0.86, *p* < 0.001), percentage of the most common response (*r* = 0.85, *p* < 0.001) and scorer 1’s accuracy judgement (*r* = −0.55, *p* < 0.05), but not scorer 2’s (*r* = −0.35, *p* = 0.13).

In French, our scorers were in a moderate agreement (*k* = 0.45). Entropy correlated significantly with both scorers’ accuracy judgements (s1 *r* = −0.87, *p* < 0.001; s2 *r* = −0.76, *p* < 0.001), number of different pronunciations (*r* = 0.92, *p* < 0.001), and percentage of the most common response (*r* = −0.85, *p* < 0.001).

In German, Entropy correlated significantly with number of different pronunciations (*r* = 0.89, *p* < 0.001), percentage of the most common response (*r* = −0.92, *p* < 0.001) but not with accuracy judgement (*r* = 0.08, *p* = 0.74). Intercorrelations can be seen in Tables 17–19.

**Table 17:** Intercorrelations for English adults reading dissimilar pseudowords (Exp 4).

Measure	1	2	3	4	5
1. Entropy	-	-.34	-.55*	.86*	-.85*
2. acc_s2	-.34	-	.54*	-.55*	.12
3. acc_s1	-.55*	.54*	-	-.75*	.22
4. n_asw	.86*	-.55*	-.75*	-	-.51*
5. perc	-.85	.12	.22	-.51*	-
n = 20					

Note: n_asw = number of different pronunciations per pseudowords, acc_s1 and acc_s2 = accuracy scored by scorer 1 and 2, perc = percentage of the most common response,

* = significant result.

**Table 18:** Intercorrelations for French adults reading dissimilar pseudowords (Exp 4).

Measure	1	2	3	4	5
1. Entropy	-	-.76*	-.87*	.92*	-.96*
2. acc_s2	-.76*	-	.58*	-.63*	.82*
3. acc_s1	-.87*	.58*	-	-.78*	.86*
4. n_asw	.92*	-.63*	-.78*	-	-.80*
5. perc	-.96*	.82*	.86*	-.80*	-
n = 20					

Note: n_asw = number of different pronunciations per pseudowords, acc_s1 and acc_s2 = accuracy scored by scorer 1 and 2, perc = percentage of the most common response,

* = significant result.

**Table 19:** Intercorrelations for German adults reading dissimilar Pseuodowords (exp 4).

Measure	1	2	3	4
1. Entropy	-	.08	.89*	-.92*
2. acc	.08	-	-.14	-.19
3. n_sw	.89*	-.14	-	-.70*
4. perc	-.92*	-.19	-.70*	-
n = 20				

Note: n_asw = number of different pronunciations per pseudowords, acc = scored accuracy, perc = percentage of the most common response,

* = significant result.

An analysis of real word misreadings revealed no significance difference between the three groups (p = 0.10 for the comparison between English and German, p = 0.12 for the comparison between English and French; p = 0.96 for the comparison between German and French). A list of pseudowords read as real words can be seen in Table 18 in S1 Appendix.

## General discussion

The present study used Entropy as a measure to assess participants’ reading aloud responses to pseudowords in English, French, Italian and German adults and children. Our main aim was to assess the impact of age, orthographic depth and bilingualism on Entropy, defined as the number of alternative pronunciations that participants give to a given pseudoword.

### The role of children’s development in Entropy

Experiment 2 clearly showed a significant decrease in Entropy (H) from grade 2 to 4, with a great fall between grade 2 and 3. This finding is in line with similar results reported in English by [12]), who show that by the end of grade 2, children already start to develop sensitivity to context-sensitive correspondences, which is probably the cause of the reduction in response variability, as Treiman and Kessler [60] suggest for spelling. In fact, children may use the surrounding context of a grapheme to derive pronunciation. This progressive diminution also explains why the majority of pronunciations by adult participants had an Entropy value of zero or very close to zero. However, data from Experiments 1, 2, 3 and 4 show that specific alternative readings did not disappear from childhood into adulthood.

Our results suggest that the pronunciation of some sublexical units is intrinsically ambiguous and variability thus does not depend on reading skills. For example, both French adults and children were divided in whether or not to pronounce final consonants that are normally silent in real words. For example, the pronunciation of a real word like “mot” (word) would uniformly be read as /mo/, while our participants read the pseudoword <stort> as /stɔr/ or /stɔrt/. Similarly, both German adults and children devoiced pseudoword codas half of the time, although final consonant devoicing is the norm in real word reading: <Bad> (bath) will always be read as /ba:t/, while the pseudoword <gund> was read as /gund/ or /gunt/. This phenomenon is not only restricted to position-sensitive correspondences, but also to context-sensitive correspondences. In German, for example, the letter <s>, followed by the letter <p>, should give the phoneme /ʃ/ as in the word “Sport” /ʃpɔrt/. Nonetheless, the pseudoword <sprau> is read by children and adults as /sprau/ or /ʃprau/. Similar instances were found in all languages, and can be found in the tables of the S1 Appendix.

### Entropy differences across languages with varying levels of orthographic transparency

To assess how the response variability to pseudowords changed in a deep compared to a shallow orthography, we tested participants in four languages that are on different points of the orthographic depth space: English, French, German and Italian.

In Experiment 1, we firstly compared English and German adults reading monosyllabic pseudowords matched on the number of letters, orthographic neighborhood and body consistency, but against our hypothesis, we did not obtain significant differences. We hypothesized that cross-linguistics differences may be manifested in childhood but would disappear into adulthood. Hence, in the subsequent experiments, we assessed cross-linguistics differences in children.

In Experiment 2 bilingual English/German children read items in both languages, but we did not find an effect of orthographic depth within the same participants. We reasoned that bilingualism itself could have caused this result, because the knowledge of one shallow orthography could have had a facilitatory effect on the deeper orthography by providing a better understanding of the systematicity of GPCs [30].

In Experiment 3 we compared Italian and French children reading a set of cognate pseudowords, against our predictions, we found that Italian children showed significant higher Entropy values than French children. However, we suspected that the reasons behind this result were to be found in the nature of the languages itself and in the items characteristics. In fact, French is considered to be asymmetric in its orthographic depth: while spelling is considered to be hard (/mɛʀ/ can be spelled as “maire” [mayor], “mère”[mother] or “mer” [sea]), reading, in spite of the presence of complex correspondences, is considered predictable [61]. Given that in a complex but predictable orthography, the pronunciation is not ambiguous, there should be a consensus in the responses.

As we did not find higher Entropy in French (a complex, predictable orthography) than Italian (a less complex, predictable orthography), this could, in theory, suggest that Entropy may not be affected by complexity. This would be a first behavioral finding suggesting that complexity and unpredictability have different effects on reading processing, thus providing further weight to the proposal of treating orthographic depth as a multidimensional construct [28]. However, the present study as it is cannot exclude with certainty that other confounding factors are not in action.

In fact, another possibility is that the items that we used in Experiment 3 may have not been representative enough of French orthography. Since we derived pseudowords from a set of cognate items which are, by definition, similar in both orthographies, they could have been lacking the presence of those complex correspondences that French and Italian do not share, but that are common in the respective languages. The CV structure of the items in French was easy, relative to the CV structure of French words in general, which may have facilitated sublexical processing in French relative to Italian and lead to the counter-intuitive finding of higher Entropy in Italian than French.

To rule out this hypothesis we administered a fourth reading aloud experiment in three groups of adults (French, German and English) using two different item sets: in the first set, items were truly representative of the three different orthographies and matched on base-word frequency, while the second set was consisted of items that were equally dissimilar in all three orthographies and had no orthographic neighbors. In the first condition, significant differences were found between English and German and English and French, but not between French and German. In the second condition significant differences were found between all three groups, with English having significant higher Entropy values than both German and French, and German having significantly higher Entropy values than French.

The findings from Experiment 4 suggest that cross-linguistic differences in Entropy seem to be a response to item characteristics [62], and in cross-linguistics research, matching pseudowords on several aspects may hide significance differences in reading behaviour. This would explains why the comparison between English and German was significant in Experiment 4, but not in Experiment 1. In Experiment 1, German and English adults read monosyllabic pseudowords matched on number of letters, orthographic neighborhood, body-neighborhood and, importantly, body consistency. The lack of a difference suggests that participants reading in English did not have overall greater uncertainty when items are made of consistent bodies. However, when English-speaking participants are confronted with a set of items truly representative of their language, with both consistent and inconsistent bodies, uncertainty arises significantly, compared to other languages.

Results from Experiment 2 seemed to follow the same direction. The use of bilingual children had the advantage that the same children read the same items in two different orthographies, thus reducing between-subject variance. While knowledge of a second orthography may have affected the results (knowing a shallow orthography—i.e. German—may have reduced the pronunciation variability of English pseudowords), we found no differences between bilinguals and monolinguals, suggesting that cross-language contamination is an unlikely explanation of the lack of a cross-linguistic difference.

### Relations among Entropy and other measures

Throughout the study we compared Entropy with the number of pronunciations per pseudowords, the percentage of the most common response and the accuracy measure. For the first two, we found, as we expected, significant positive correlations between Entropy and number of pronunciations. Clearly, as the number of pronunciations increase, Entropy values also increases. At the same time, the higher the percentage of the most common response to a pseudoword is, the lower the Entropy value for that particular item is, since a high percentage of the most common response means that participant strongly agreed on the pronunciation. Therefore, Entropy and percentage of the most common response was always in a negative, significant correlation.

The most interesting relationship was found between Entropy and accuracy. For three of the four language groups (Italian, German and French), we asked two different scorers who had received training in the phonology of the respective language to evaluate the accuracy of pseudoword readings. We calculated Cohen’s kappa to determine scores’ agreement. Strong agreement was found in bilingual children reading English items (Exp 2), French children (Exp 3) and English-speaking participants reading dissimilar pseudowords (Exp 4), although we found a nearly perfect agreement only in Italian scorers. Strong agreements were found across all children: a possible explanation would be that judging children’s response accuracy was easier for scorers, as some readings were clearly not plausible. Regrettably, we could not hire a second scorer for the German data to provide further evidence to this hypothesis. Since our grade comparison focused on German data, it is possible we could find a negative correlation between scorers’ agreement and grade.

Strong agreement was found for English-speaking adults in Experiment 4. It seems that accuracy agreement on pronunciations in which phoneme-grapheme correspondences were clearly unlikely (“gist” read as “gust, <i> − > /u/) is higher compared to the accuracy agreement on pronunciations where readers have to decide which phonemes, virtually associated to a particular grapheme, are to choose (“gid” read as /gid/ or /ʤist/). Since dissimilar English pseudowords were associated with the greatest number of different pronunciations in all three groups, it is not surprising that scorers found a strong agreement in this group as well, even if the participants were adults.

The only nearly perfect agreement was found in Italian data: this may be due to age, because participants were children, and to the fact that Italian is the most transparent language in the pool. This suggests that accuracy and Entropy were not correlated in Italian, because the number of implausible readings was very low, and Entropy was driven by the presence of two or more plausible pronunciations. For example, in Italian there are two phonemes mapping to <g>: but scorers marked both pronunciations as correct based on a lenient marking criterion.

All in all, while by definition Entropy should be significantly, and negatively correlated with accuracy, in practice accuracy judgement itself, for pseudowords, is not a straightforward and error-free process. Even though we gave the same instructions to all scorers, and even though all scorers were trained in phonology, there is variability in judgement both between scorers and within scorers (as the results for the Monolingual German children sample seem to show).

We interpret this finding as a further evidence that accuracy scoring is subject to arbitrariness and its reliability is low. Accuracy, as a measure to evaluate pseudoword reading behavior, is less than ideal. This finding points toward the need to find a different, subjectivity-free measure to investigate pseudoword reading aloud behavior. Since Entropy calculation does not involve any type of human intervention and is a complete, mathematical process, we propose here that Shannon’s Entropy, when investigating item-level behavior, could represent, in this regard, a good candidate.

### Limitations and future directions

In this study we isolated the effects of orthographic depth, age and bilingualism on a new measure for pseudowords reading aloud performance: Entropy. This measure opens possibilities for future research. The relationship between this measure and a more traditional one, reaction time requires further clarification. For example, pseudowords associated with high Entropy values can take more time to read, because readers have to scan all plausible phonological representations and decide which one is more fitting given a particular context. This would shed light about the cognitive processes that correlate with pseudoword reading aloud Entropy. It is possible that readers have a set of context-sensitive GPCs which they always apply when they encounter a particular orthographic cluster. It might depend on their reading experience, and in particular the frequency with which they encounter a given cluster in real words. Activation of other possible pronunciations is suppressed at an early processing stage, such that Entropy is not reflected in participants’ response latencies. Alternatively, it is possible that participants generate possible pronunciations at a late processing stage, before articulation is initiated. This would lead to a closer link between item-level Entropy and RT.

An advantage of the Entropy measure is that its calculation is theory-neutral. While we used the terminology of the Dual Route Cascaded model throughout the paper (e.g. grapheme-phoneme correspondences), the results also fit within alternative models of reading, such as Connectionist models [63, 64]. The current analysis do not allow us to provide evidence for one model over another.

However, this could be a direction for future research. For example, language-level Entropy at different unit sizes (as described by [18] could be used as a predictor of pseudoword reading aloud Entropy. This would allow us to assess whether GPCs, as currently implemented in the DRC, are the best predictors of Entropy, or if participants rely on larger units such as bodies. Our multiregression analysis seem to suggest, already, that variables such as orthographic neighborhood, phonological neighborhood, body neighborhood, baseword and bigram frequency and number of syllables do not seem to be good predictors. A further possibility would be to investigate the role of sublexical units in Entropy: using participant-level Entropy by presenting the same participants repeatedly with the same orthographic units (as was done by [12]), would allow us to assess whether participants use the same type of units across time, or if there is intra-participant variability in which type of correspondence is applied, which would speak against the notion of an all-or-none rule.

Methodologically, future research on the application of the Entropy measure in pseudowords reading behavior may want to directly assess how and if the present findings change given a different sample size. As with all measures, Entropy is likely to be sensitive to sample size: smaller samples are more likely to be affected by random noise. Furthermore, the number of possible pronunciations, which is a major determiner of the Entropy measure, depends on the number of participants, because the maximum number of possible pronunciations is capped by the number of participants. In practice, the number of different pronunciations is likely to be lower than the number of participants in our experiments. For example, Pritchard, Coltheart, Palethorpe and Castles [7] found, on average, 8 different responses among 45 English-speaking participants. Nevertheless, future research is required to establish at what sample sizes and under what circumstances pseudoword reading aloud Entropy yields stable estimate.

Finally, in our fourth experiment, we randomly picked base words from which we derived pseudowords, without any systematic control (exception made for frequency) to linguistic properties such as body neighborhood, orthographic neighborhood, or number of syllables and length (for the first subset). Our choice was driven by the consideration that we could not find significant cross-linguistics differences in Entropy using systematically chosen items that matched across languages in Experiment 1-3, and we suspected that the item characteristics themselves could be the cause. Although choosing items whose orthographic characteristics were controlled for has the advantage of having potentially psycholinguistic relevant factors contained (for example, the word length), in cross-linguistic research using items that were forced to be similar across orthographies may prevent an adequate representation of the different orthographies features of the languages in question [49]. This shortcoming could be avoided by choosing random base words from which we can derive pseudowords, while using only frequency as a control variable (since frequency is not orthography related, contrary to the above-mentioned characteristics). However, a random selection of base words can result in an unbalanced list, merely due to chance rather than as a reflection of the systematic features of the language. To account for both deficiencies, we decided to try both approaches.

Another interesting application of the Entropy measure may be to investigate subject-level performances. In our study we used Entropy to assess item-level variability while averaging across participants. However, more could be done, for example, by using Entropy to calculate intra-participant variability (whether the same participant was consistent in pronunciation when asked to read a given pseudoword more than once). Lastly, in the current study we investigated how Entropy correlated with accuracy and discussed the short-comings of the latter in pseudoword reading behavior investigation. However, some questions are still unresolved. It remains to ascertain if and how Entropy interacts with other measures, such as reaction times: while pseudoword accuracy scoring is subject to human arbitrary decisions, RT measures are not. At the same time, in our study we did not focus on what specifically could be a predictor of Entropy.

As an exploratory analysis, we ran some multiple regressions addying body neighborhood, orthographic neighborhood, baseword frequency, bigram frequency, number of syllables and length as predictors (forin Experiment 1, 2 and 4), but none of those turned out to be reliable predictors (length only affected Entropy in the comparison between English and French). Future studies could look into this specifically, for example by running a model using different measures such as body-rime consistency or vowel consistency, like in [18].

## Conclusion

The present study contributes to the literature using Entropy as a measure to quantify the variability in pseudowords pronunciation. We investigated whether Entropy changes in relationship to orthographic depth, age and bilingualism.

The results indicate that deeper orthographies lead to higher Entropy values, provided that items are truly representative of the orthographies under scrutiny. Furthermore, our preliminary results suggest that the effect of Entropy is driven by the degree of unpredictability, but not by the complexity of an orthography. This is a first demonstration of a differential effect of complexity and unpredictability as dissociable constructs underlying orthographic depth, which stresses the need to consider the multidimensional nature of orthographic depth in cross-linguistic reading research.

The present study demonstrates that Entropy decreases across age, indicating that the agreement on pseudoword pronunciations increases in relation to the development of reading skills. Finally, we did not find significant differences in Entropy values between monolingual and bilingual children. All this considered, this study can be regarded as a starting point to evaluate the use of alternative measures, and specifically Entropy, to investigate cross-linguistics differences in pseudoword reading and reading development.

## Supporting information

S1 Appendix (PDF)
