# Supplementary material for: Order among chaos: Cross-linguistic differences and developmental trajectories in pseudoword reading aloud using pronunciation Entropy
Source: PLoS One. 2021 May 19;16(5):e0251629. doi: 10.1371/journal.pone.0251629 (PMC8133407; doi:10.1371/journal.pone.0251629)
Supplement: S1 Appendix — (PDF) [file pone.0251629.s001.pdf]

S1 Appendix

Table 1. List of pseudowords read as real words (Exp 1).

Pseudoword	Word	Nread	Translation	Percentage	Participants
stide	tag	1/24		4.17	English
floud	flood	1/24		4.17	English
dinn	dünn	1/19	thin	5.25	German
reuz	kreuz	2/19	cross	10.53	German
stork	stock	1/19	floor	5.25	German
wolz	wolf	1/19	wolf	5.25	German

Note: Nread indicates the number of participants in the group that read the pseudoword as real words.

Table 2. German Adults reading matched monosyllabic pseudowords (Exp. 1)

Items	Pronunciations	Comments
quang	(6) kvaŋ, kwaŋg<br>(5) kwaŋ (1) kwan, kvaŋg	Different readings for the grapheme <u>: /v/ or /w/<br>Different reading for the grapheme <n[g]>: /ŋ/ or /ng/
splur	(10) ʃplur, (4) splur, (2) ʃplu:r,<br>(1) splu:r, ʃplul, plu:r	Different readings for the grapheme <s[p]>: /ʃ/ or /s/<br>Different vowel lengths + Phoneme replacement or deletion
frur	(11) fru:r, (5) frur,<br>(1) flu:r, flu:rur, fur	Different vowel lengths + Phoneme deletion and replacement<br>Addition of syllable (flu:rur)
gund	(9) gunt, (7) gund,<br>(2) gu:nd, (1) grund	Real word reading (grund - reason)<br>The final consonant devoicing rule is either applied or not<br>Different Vowel lengths
mang	(9) maŋ, (9) maŋg, (1) maŋk	Different readings for the grapheme <n[g]>: /ŋ/, /ŋg/ or /ŋk/
schweck	(11) ʃvek, (5) svek,<br>(2) ʃveŋk, (1) ʃve:k	Different vowel lengths + phoneme insertion (ʃveŋ k)<br>Different readings for the grapheme <s>: /ʃ/ or /s/
zein	(9) zain, (8) tsain,<br>(1) tse:n, tsaim	Different readings for the following graphemes:<br><z> as /ts/ or /z/ and <ei> as /ai/ or /e:/
zwau	(9) tsvau, (8) zvau,<br>(1) tsva:u, zvau:	Different readings for the grapheme <z>: /ts/ or /z/<br>Different vowel lengths
sprau	(13) ʃprau, (3) sprau,<br>(2) ʃpau, (1) ʃprau:	Different readings for the grapheme <s[p]>: /ʃ/ or /s/<br>Different vowel lengths + Phoneme deletion (ʃpau)
fold	(14) folt, (2) fold, fo:lt<br>(1) foalt	The final consonant devoicing rule is either applied or not<br>Different vowel lengths

Note: The numbers in parentheses indicate how many participants read the item with the following pronunciations

Table 3. English Adults reading matched monosyllabic pseudowords (Exp. 1)

Items	Pronunciations	Comments
wurn	(11) wɛ:n, (2) wɛrn<br>(1) wuʌn, wʌn, wun,<br>(1) wo:n, woʌn, w3:nt	Different readings for the following graphemes:<br><u[r]> as /ɛ/, /u/, /a/, /o/ and <[u]r> as /ʌ/ or ø
dize	(14) daiz, (2) dizi,<br>(1) di:z, di:ze, dizi	Different readings for the following graphemes<br><i>: as /ai/ or /i/ and final <e> as /i/, /e/ or ø
gule	(14) gu:l, (2) gju:l, g ʌl, (1) goʊl	Different readings for the grapheme <u>: /u/, /oʊ/, /ʌ/
kuy	(10) kai, (6) kui, (2) ki:, (1) ku:	Different readings for the grapheme <uy>: /ai/, /i:/, /u:/ or /ui/
luice	(10) luis, (6) lu:s, (3) lus	Different readings for the grapheme <ui>: as /u:/, /u/ or /ui/
whun	(14) wʌn, (2) wun<br>(1) huan, hwʌn, wu:n	Different readings for the following graphemes:<br><u> as /u/, /u:/ or /ʌ/ and <w> as /h/ or /w/
pluit	(13) pluit, (5) plu:t, (1) plait	Different readings for the grapheme <ui>: /ui/, /u/, /ai/
sirt	(13) sɛ:t, (2) sɛʌt, sɛt,<br>(1) siʌt, sɛ:ʌt	Different readings for the following graphemes:<br><[i]r> as /ʌ/ or øand <i[r]> as /ɛ/, /ɛ:/ or /i/
fice	(12) fais, (4) fi:s	Different readings for the grapheme <i>: /ai/ or /i:/
yorch	(16) jo:ʧ, (1) jorʧ, jortn, jorʧt	Different readings for the grapheme <[o]r>: /ʌ/ or ø<br>Phoneme addition (jorʧt)

Note: The numbers in parentheses indicate how many participants read the item with the following pronunciations

Table 4. Bilingual German/English children (grade 2) reading English-like pseudowords (Exp. 2)

Item	Pronunciations	Comments
derge	(3) dɜ:ʤ<br>(1) deɹʤ, dik, dɜɹg, dɜ:ge,<br>dɜɹʤ, dɜ:rg, dɹeg, dɜ:g, deɹg	Different readings for the following graphemes:<br><e[r]> as /ɜ:/ or /e/ and <g> as /ʤ/ or /g/
gurt	(4) gɜ:t<br>(2) gʌ:t<br>(1) gɜʌt, got, goɹt, gæɔt, goʊɹt	Different readings for the following graphemes:<br>u[r] as /ɜ:/, /ʌ:/, /ɜr/, /oʊ/, /oɹ/<br><u> as /æɔ/, /ʌ/
gule	(4) gu:l,<br>(2) gʌl<br>(1) gul, glu:, glai, goʊl, gju:l	Different readings for the grapheme <u>: /u/, /oʊ/, /ju/<br>Different vowel lengths<br>Real word reading (glue)
murse	(5) mɜ:s<br>(2) moɹs<br>(1) muɹze, mɜʌs, mɜ:si, maɹs	Different readings for the following graphemes:<br><u[r]> as /ɜ:/, /ʌ/, /ɜɹ/, /o/ and <s> as /z/ or /s/
luice	(5) luis<br>(1) lauis, lu:s, luls, lak, laŋk	Different readings for the grapheme <u[i]>: /u:/ or /ui/<br>Phoneme insertions (luls, laŋk)
pluit	(5) plu:t<br>(2) pluit, plinkt, plait, plot, plont	Different readings for the grapheme <u[i]>: /u:/ or /ui/<br>Phoneme insertions (plinkt, plont)
suzz	(6) sʌz<br>(1) su:z, suts, sʌts, zʌz, suz	Different readings for the following graphemes:<br><z> as /z/ or /ts/, <s> as /z/ or /s/ and <u> as /ʌ/ or /u/<br>Different vowel lengths
sirt	(5) sɜ:t<br>(4) siɹt<br>(1) zɜ:t, ʃɜ:t, sɜɹt	Different readings for the following graphemes:<br><s> as /ʃ/, /z/ or /s/ and <i[r]> as /ɜɹ/ or /iɹ/
tirm	(5) tɜ:m (3) tɜɹm<br>(1) tɹim, tim, tiɹm	Different readings for the following graphemes:<br><i[r]> as /ɜ/, /i/, /ɜ:/ and <[i]r> as /ɹ/ or /ø/
roud	(6) ɹæ<br>(2) ɹu:d, ɹæɔnd<br>(1) ɹoʊd, ɹod	Different readings for the following grapheme:<br><ou> as /æɔ/, /u:/, /oʊ/, /o/<br>Real word reading (round)

Note: The numbers in parentheses indicate how many participants read the item with the following pronunciations

Table 5. Bilingual German/English children (grade 3) reading English-like pseudowords (Exp. 2)

Items	Pronunciations	Comments
derge	(2) dɛːʤ,<br>(1) dɛrʤ, dɹag, dɛːg	Different readings for the following graphemes:<br><[e]r> as ɹor øand <g> as /g/ or /ʤ/<br>Phoneme inversion (drag)
fich	(2) fiʧ,<br>(1) fiʃ, fɹiʃ, fit	Different reading for the grapheme <ch>: /ʃ/ or /ʧ/<br>Real word reading (fish)<br>Phoneme insertion (friʃ)
gule	(2) gʌl,<br>(1) gu:l, gju:l, glu:	Different reading for the grapheme: <u> as /u:/, /ʌ/ or /ju/<br>Real word reading (glue)
krilk	(2) kɹilk,<br>(1) kɹik, klɛ:k, kɹai	Different readings for the grapheme <i>: /i/, /ai/ or /ɛ:/<br>Phoneme deletion (kik)
pliz	(3) plits, (1) plis, pliz	Different readings for the grapheme <z>: /ts/, /z/ or /s/
pluit	(2) plu:t,<br>(1) plu:ʧ, pluit, palt	Different readings for the grapheme <ui>: /u:/ or /ui/<br>Phoneme inversion (plat)
stum	(2) stam,<br>(1) stʌŋ, stu:m, stum	Different readings for the following graphemes:<br><u> as /ʌ/, /u:/ or /u/ and <m> as /ŋ/ or /m/
whun	(2) wʌn,<br>(1) win, wu:m, wu:n	Different readings for the following graphemes:<br><u> as /ʌ/, or /u:/ and <n> as /m/ or /m/
wrum	(2) ɹam,<br>(1) wan, wɛ:n, wo:m	Different readings for the following graphemes:<br><u> as /ʌ/, /ɛ:/ or /o:/ and <m> as /n/ or /m/
chyle	(2) kai, ʧail, (1) cycle	Different readings for the grapheme <ch>: /ʧ/ or /k/

Note: The numbers in parentheses indicate how many participants read the item with the following pronunciations

Table 6. Bilingual German/English children (grade 4) reading English-like pseudowords (Exp. 2)

Items	Pronunciations	Comments
gule	(1) ʤu:l, glu:, gu:l, gʌl, gju:l	Different readings for the following graphemes:<br><u> as /u:/, /ʌ/ or /ju:/ and <g> as /g/ or /ʤ/
whun	(2) wʌn,<br>(1) wu:n, vʌn, wun	Different readings for the following graphemes:<br><u> as /u:/, /u/ or /ʌ/ and <w> as /w/ or /v/
yorch	(2) yo:ʧ,<br>(1) zoɹʧ, youɹʧ, ʧoɹʧ	Different readings for the grapheme <[o]r>: /ɹ/ or ø<br>Phoneme replacement (ʧoɹʧ)
barsh	(2) ba:ʃ, baɹʃ,<br>(1) bɹaʃ	Different readings for the grapheme <[a]r>: /ɹ/ or ø<br>Phoneme inversion (bɹaʃ)
chycle	(2) ʧail, cycle (1) kju:li	Different readings for the grapheme <ch>: /ʧ/ or /k/
splaw	(2) splo:, splæɔ, (1) spo:	Different readings for the grapheme <aw>: /o:/ or /æɔ/
swuff	(2) swʌf, swuf,<br>(1) stuf	Different readings for the grapheme <u>: /u/ or /ʌ/<br>Phoneme replacement (stuf)
wrum	(2) ɹam, wɛ:m,<br>(1) woɹm	Different readings for the following graphemes:<br><u> as /ʌ/ or /ɛ:/ and <w> as /w/ or ø
chy	(3) ʧi,<br>(1) ʧi:, ʃai	Different readings for the following graphemes:<br><y> as /i:/, /i/ or /ai/ and <ch> as /ʧ/ or /ʃ/
frict	(3) fɹikt, (1) fikt, fɹiʧ	Phoneme deletion (fikt)

Note: The numbers in parentheses indicate how many participants read the item with the following pronunciations

Table 7. Bilingual German/English children (grade 2) reading German-like pseudowords (Exp. 2)

Items	Pronunciations	Comments
splur	(3) ʃplu:r, ʃplur<br>(2) ʃplʊ:r<br>(1) splu:r, splʊr, ʃplu:ur, ʃpu:ur, ʃpau	Different readings for the grapheme <s>: /ʃ/ or /s/,<br>even if <s>before <p>should always be ʃ<br>Different readings for the grapheme <u>: /u/ or /ʊ/<br>Different vowel lengths, Phonemes deletion
reuz	(6) roits,<br>(1) roiz, raits, rʊts, kroits, krets, raiz, ru:z, røz	Different readings for the diphtong <eu>: /oi/, /ai/, /e/ and /ø/<br>Different readings for the grapheme <z>: /z/ or /ts/<br>Real word reading (kreuz - cross)
klund	(5) klunt,<br>(2) kluŋk<br>(1) kolt, klaunt, klu:nt, klun, klund	The final consonant devoicing rule is either applied or not<br>Real word reading (colt)<br>Different readings for the grapheme <n>: /ŋ/ or /n/<br>Different vowel lengths, phoneme deletions
goos	(4) gu:s,<br>(3) gus, gos,<br>(1) gʊ:s, gʊs, bu:s	Different readings for the grapheme <oo>: /u/, /o/ or /ʊ/<br>Real word reading (bus)<br>Different vowel lengths
frur	(5) fru:r,<br>(2) frør, frur,<br>(1) fu:r, jur, frau:ç	Different readings for the grapheme <u>: /u/ or /ø/<br>Different vowel lengths, phonemes deletions and replacements
lonch	(5) lœn:ç,<br>(2) loinç, loç,<br>(1) lʊnç, lʊnf, lʊk	Different readings for the grapheme <o>: /o/, or /ʊ/<br>Different readings for the grapheme <ch>: /ç/ or /k/<br>Phoneme deletions
seng	(5) zeŋ, zeŋk,<br>(1) zain, ze:ŋk, ziŋ, zin	Different readings for the grapheme <n>: /n/ or /ŋ/<br>Different readings for the grapheme <e>: /e/, /ai/ or /i/<br>Final consonant devoicing or deletion
pang	(5) paŋk,<br>(2) beŋ, paŋ,<br>(1) praŋ, paŋg, pa:ŋg	Different readings for the grapheme <n>: /n/ or /ŋ/<br>The final consonant devoicing rule is either applied or not<br>Different vowel lengths<br>Phonemes replacement, deletions or insertion
truck	(6) bruk,<br>(3) truk,<br>(4) brʊke, bru:k, gral, brʊk	Different readings for the consonant cluster <tr>: /br/ or /tr/<br>Different readings for the grapheme <u>: /u/ or /ʊ/<br>Different vowel lengths + Phonemes replacement
spand	(5) ʃpant, (3) spant,<br>(2) spand, (1) swant, ʃpand	Different readings for the grapheme <s>: /ʃ/ or /s/,<br>even if <s>before <p>should always be ʃ<br>The final consonant devoicing rule is either applied or not

Note: The numbers in parentheses indicate how many participants read the item with the following pronunciations

Table 8. Bilingual German/English children reading German-like pseudowords in grade 3 (Exp. 2)

Items	Pronunciations	Comments
beld	(1) belt, be:lt, pelt, blent	Final consonant devoicing<br>Real word reading (blend - it dazzles)<br>Different vowel lenghts + Phoneme inversion and insertion
frur	(2) frur,<br>(1) fu:r, frʊr, fru:r	Different readings for the grapheme <u>: /u/ or /ʊ/<br>Different vowel lengths + Phonemes deletion
pies	(2) pi:s,<br>(1) pa:is, pis, bi:s	Different readings for the grapheme <ie>: /i/ or /ai/<br>Probable recognition of the English word “pies” and subsequent reading.<br>Different vowel lenghts + Phoneme replacement
poot	(2) po:t,<br>(1) pu:t, plut, pot	Different readings for the grapheme <oo>: /o/ or /u/<br>Different vowel lengths + Phoneme insertion
reil	(2) rail, (1) ra:ail, pail, prail	Phoneme insertions
splur	(2) ʃplur, (1) ʃplu:r, ʃlur, splur	Different readings for the grapheme <s>: /ʃ/ or /s/,<br>even if <s> before <p> should always be ʃ<br>Different vowel lengths + Phonemes deletions
lusch	(2) luʃ, lu:ʃ,<br>(1) loʃ	Different readings for the grapheme <u>: /u/ or /o/<br>Different vowel legths
melz	(2) melz, melts,<br>(1) molz	Different readings for the grapheme <z>: /z/ or /ts/<br>Phoneme replacement
kreck	(3) krek, (1) frek, ʃrek	Real word readings (frech - rebellious & schreck - fright)
quang	(3) kwaŋ (1) kwaŋk, kuwaŋ	Final consonant devoicing<br>Phoneme deletion and insertion

Note: The numbers in parentheses indicate how many participants read the item with the following pronunciations

Table 9. Bilingual German/English children reading German-like pseudowords in grade 4 (Exp. 2)

Items	Pronunciations	Comments
zwau	(2) tsvau, (1) zvau, tsau	Different readings for the grapheme <z>: /z/ or /ts/
spand	(2) ʃpant, (1) ʃpaŋ, ʃpand	Different readings for the grapheme <s>: /ʃ/ or /s/,<br>even if <s> before <p> should always be ʃ<br>The final consonant devoicing rule is either applied or not<br>Different readings for the grapheme <n>: /n/ or /ŋ/
retz	(2) retst, (1) pets, rets	Phoneme insertion and replacement
poot	(2) pu:t,<br>(1) put, prot	Different readings for the grapheme <oo>: /u/ or /o/<br>Possible influences from the knowledge of English<br>Different vowel lengths + Phoneme insertion
pies	(2) pi:s,<br>(1) pis, pais	Different readings for the grapheme <ie>: /i/ or /ai/<br>Probable recognition of the English word "pies" and subsequent reading.<br>Different vowel lengths
nech	(2) neç,<br>(1) neʧ, heç	Different readings for the grapheme <ch>: /ç/ or /ʧ/<br>Phoneme replacement
jenf	(2) ʧenf, jenf	Different readings for the grapheme <j>: /j/ or /ʧ/
laat	(2) lat, la:t	Different vowel lengths
mohl	(2) mol, mo:l	Different vowel lengths
silm	(2) zilm, zelm	Different readings for the grapheme <i>: /i/ or /e/

Note: The numbers in parentheses indicate how many participants read the item with the following pronunciations

Table 10. Monolingual German children (grade 2) reading monosyllabic pseudowords (Exp. 2)

Item	Pronunciations	Comments
dels	(6) delts (3) de:lts, belts (2) delt,<br>(1) dels, delz, de:lt, te:ls,<br>de:ls, de:s, dele:ts, dalts	Phoneme replacements, insertions or deletion<br>different vowel lengthening<br>different readings for the grapheme <s> (/ts/, /s/, or /z/)
silm	(9) zilm, (3) zil,<br>(2) zelm, silm<br>(1) selm, tsil, zelf, zi:l, fi:lm, tsilm	Phoneme replacements and deletions, due to mispronunciations<br>Different readings for the grapheme <s> (/ts/, /s/, or /z/), and <e> (/i/ or /e/)<br>Real word readings (film)
keiz	(9) kraits<br>(2) keits, keis, kreits<br>(1) kaints, kreis, veiz, kalts,<br>kraits, kaiz, ke:ts	Different readings for the grapheme <ei> (/ai/,/ei/ or /e:/),<br>although the first one is the correct one.<br>Different readings for the grapheme <s> (/ts/, /s/, or /z/)<br>Phoneme insertions or replacements due to mispronunciations
grein	(11) grain, (5) grai:n<br>(3) krain, (2) gren<br>(1) grai:, kre:, gre, gre:n	Different readings for the grapheme <ei> (/ai/,/ei/ or /e:/)<br>Devoicing of the first consonant <g> -> k<br>Different vowels length
gund	(10) grain, (4) grai:n, (3) krain<br>(2) gren, (1) gre:n, gre, kre:, grai:	Application or no of the final consonant devoicing phonotactic rule<br>Different vowels length, first consonant devoicing<br>Participants read similar real words instead of the item (grund - reason)<br>Phoneme deletion or insertion
quang	(7) kuaŋk, (4) kuaŋg,<br>(3) kwaŋk, kuwaŋk<br>(1) kuaŋ, kwaŋ, kuwaŋg	Different readings for the grapheme <u> (/u/ or /w/)<br>The final consonant devoicing phonotactic rule is either applied or not<br>Phoneme insertion or deletion
reuz	(8) roits, (5) kreuts,<br>(1) reuts, keuts, raits,<br>roi:z, roi:, roi:ts	Different readings for the grapheme <eu> (/oi/, /eu/ or /ai/)<br>Different readings for the grapheme <z> (/ts/ or /z/)<br>Real word readings (kreuz - cross)<br>Different vowel length
melz	(1) melts, (4) me:lts,<br>(1) mol, nelts, ne:z, ma:lts, malts	Different readings for the grapheme <z> (/ts/ or /z/)<br>Phonemes deletion or replacements<br>Different vowel lengths
seng	(8) zeŋk, (4) tseŋk, tseŋg<br>(2) tsiŋk (1) seŋk, ziŋk	Different readings for the grapheme <s> (/ts/, /z/ or /s/)<br>The final consonant devoicing phonotactic rule is either applied or not<br>Final consonant deletion
sinks	(13) ziŋk, (2) tsiŋks<br>(1) siŋkts, tsiŋkts, stiŋkz,<br>tsiŋ, tsiŋk, ziŋkz, ziŋk	Different readings for the grapheme <s> (/ts/, /z/ or /s/)<br>Phonemes insertion or deletion

Note: The numbers in parentheses indicate how many participants read the item with the following pronunciations

Table 11. Monolingual German children (grade 3) reading monosyllabic pseudowords (Exp. 2)

Items	Pronunciations	Comments
quang	(7) kwaŋg,<br>(5) kwaŋk,<br>(2) kuwaŋk, kwan, kwaŋ<br>(1) gwaŋk, kuwaŋg	The final consonant devoicing rule is either applied or not<br>Different readings for the grapheme <n>: /n/ or /ŋ/<br>even if it should always be ŋ before g<br>Different readings for the initial grapheme <g>: /g/ or /k/<br>Phoneme insertions and deletions
pang	(9) paŋk, (3) paŋg,<br>(2) peŋk, paŋ,<br>(1) pan, pa:ŋg, praŋg	The final consonant devoicing rule is either applied or not<br>Different readings for the graphene <a>: /a/ or /e/<br>Different vowel lengths + Phoneme insertions and deletions
dels	(8) delts,(5) delt,<br>(2) de:lz, delz, (1) belts	Different readings for the grapheme <s>: /z/ or /ts/<br>Different vowel lengths + Phoneme replacements
teins	(11) taints, (2) tainz, paints,<br>(1) painz, taits, tain, taint, taiŋ ts	Different readings for the grapheme <s>: /s/ or /ts/<br>Phoneme replacements
goos	(8) gos,<br>(5) go:s<br>(2) gu:s, (1) gous, gu:ʃ, gus	Different readings for the grapheme <oo>: /o/, /ou/ or /u/<br>Different radings for the grapheme <s>: /s/ or /ʃ/<br>Different vowel lengths
femd	(10) femt<br>(4) fent<br>(1) fe:nt, fem, fremt	The final consonant devoicing rule is either applied or not<br>Different readings for the grapheme <m>: /n/ or /m/<br>Different vowel lengths + Phoneme insertions and deletions
sinks	(11) ziŋks<br>(2) ziŋk<br>(1) zints, tsiŋks, ziŋkst, skiŋkz, zinks	Different readings for the grapheme <s>: /z/, /ts/ or /s/<br>Different readings for the grapheme <n>: /n/ or /ŋ/<br>Phoneme insertions and deletions
gund	(11) gunt<br>(3) gund<br>(2) gu:nt<br>(1) kult, krunt, bunt	The final consonant devoicing rule is either applied or not<br>Real word reading (bund - confederation)<br>First consonant devoicing /g/ ->/k/<br>Different vowel lengths + Phoneme replacement
reil	(8) rail, (7) krail, (2) prail, (1) grail, frail	Consonant insertion before the first consonant
grein	(11) grain, (4) krain<br>(1) grai:n, gain, graint, gwain	First consonant devoicing<br>Different vowel lengths + Phoneme insertions and deletions

Note: The numbers in parentheses indicate how many participants read the item with the following pronunciations

Table 12. Monolingual German children (grade 4) reading monosyllabic pseudowords (Exp. 2)

Items	Pronunciations	Comments
quang	(6) kwaŋk	The final consonant devoicing rule is either applied or not
	(5) kwaŋ	Different readings for the grapheme <n>: /n/ or /ŋ/
	(4) kwaŋg	even if it should always be ŋ before g
	(3) kuwaŋk	Different readings for the grapheme <a>: /a/ or /e/
	(1) kwant, praŋk, kweŋk	Phoneme insertions, deletions and replacements
pehl	(11) pe:l, (4) pel	Different vowel lengths + Phonemes insertions and deletions
	(2) perl, fe:l, (1) pe, pfe:l	Real word readings (Perl & Fehl - flaw)
beld	(13) belt, (3) be:lt	The final consonant devoicing rule is either applied or not
	(2) delt	First consonant devoicing /b/ ->/p/
	(1) beld, pelt, berlt, telt	Different vowel lengths + Phoenemes insertion ands replacements
pang	(11) paŋ, (5) paŋk	The final consonant devoicing rule is either applied or not
	(4) pan	Different readings of the grapheme <n>: /n/ or /ŋ/
	(1) klak, paŋg	Phonemes deletions and replacements
seng	(9) zeŋ, zeŋk	The final consonant is either devoiced or not read
	(1) zen, reŋ	Different readings for the grapheme <e>: /e/ or /i/
	(2) ziŋ	Different readings of the grapheme <n>: /n/ or /ŋ/
goos	(10)go:s, (8) gos	Different readings for the grapheme <oo>: /o/ or /u/
	(2) gus, (1) bos, fo:s	Different vowel lengths + Phonemes replacements
drast	(12) drast	First consonant devoicing /d/ ->/t/
	(5) dra:st	Different readings for the grapheme <a>: /a/ or /e/
	(2) trast, (1) tra:st, drest	Different vowel lengths
reuz	(11)roits	Different readings for the diphtong <eu>: /oi/, /au/
	(6) kroits	Different readings for the grapheme <z>: /s/ or /ts/
	(1) kraus, poits, rois	Real word reading (kreuz - cross)
		Phonemes replacements and insertions
kust	(15) kust, (3) ku:st	Real word reading (kunst - art)
	(1) kus, gust, kunst, kuts	Different vowel lengths + Phonemes deletions and replacement
mang	(10) maŋ, (9) maŋk	The final consonant devoicing rule is either applied or not
	(2) maŋg, (1) moŋ	Different readings for the grapheme <a>: /a/ or /o/

Note: The numbers in parentheses indicate how many participants read the item with the following pronunciations

Table 13. List of pseudowords read as real words in Experiment 2 (Bilingual children).

Pseudoword	Word	Nread	Translation	Percentage	Items	grade
roud	round	2/12		16.67	en	two
gule	glue	1/5		20	en	two
roud	round	2/5		40	en	two
traw	straw	1/4		25	en	three
roud	round	1/5		20	en	four
waus	raus	1/14	outside	7.13	de	two
mauch	maus	1/12	mouse	8.32	de	two
gund	grund	1/12	reason	8.32	de	two
krein	klein	1/12	small	8.32	de	two
reuz	kreuz	1/13	cross	7.7	de	two
truck	brücke	1/13	bridge	7.7	de	two
wolz	wolf	1/14	wolf	7.13	de	two
wolz	volt	2/14	voltage	14.29	de	two
polf	pol	2/11	pole	18.17	de	two
gund	grund	1/10	reason	10	de	two
plur	pur	1/12	pure	8.32	de	two
laat	laut	1/13	loud	7.7	de	two
pies	pies	3/11	cakes	27.26	de	two
truck	brücke	1/13	bridge	7.7	de	two
wolz	volt	1/13	voltage	7.7	de	two
wolz	wolf	1/13	wolf	7.7	de	two
laft	lauf	1/5	run	20	de	three
pies	pies	1/4	cakes	25	de	four

Note: Nread indicates the number of participants in the group that read the pseudoword as real words.

Table 14. List of pseudowords read as real words in Experiment 2 (Monolingual children).

Pseudoword	Word	Nread	Translation	Percentage	grade
frur	Frau	1/16	madame	6.25	two
gund	gut	1/19	good	5.25	two
gund	Grund	2/19	reason	10.53	two
jaus	Haus	1/19	house	5.25	two
kast	Gast	1/19	guest	5.25	two
femd	fremd	1/17	foreign	5.89	two
kast	Gast	1/19	guest	5.25	three
kast	krass	1/19	great	5.25	three
krau	grau	1/19	grey	5.25	three
dinn	dünn	1/19	thin	5.25	three
femd	fremd	1/19	foreign	5.25	three
kust	Kunst	2/19	art	10.53	three
reuz	Kreuz	3/17	cross	17.65	three
polf	Golf	1/22	golf	4.54	four
polf	Wolf	1/22	wolf	4.54	four
frur	fur	1/20	for	5	four
gund	Grund	1/22	reason	4.54	four
kast	Gast	1/22	guest	4.54	four
krau	Kraut	1/22	herb	4.54	four
femd	fremd	1/21	foreign	4.75	four
kust	Kunst	1/22	art	4.54	four
reuz	Kreuz	6/22	cross	27.26	four

Note: Nread indicates the number of participants in the group that read the pseudoword as real words.

Table 15. List of pseudowords read as real words in Experiment 3

Pseudoword	Word	Nread	Translation	Percentage	Participants
orrivo	arrivo	4/32	arrival	12.5	Italian
amdio	amido	6/32	starch	18.75	Italian
antobus	autobus	5/32	autobus	15.62	Italian
benge	bende	1/32	bandage	3.13	Italian
calion	camion	1/32	truck	3.13	Italian
clampagne	campagne	1/32	champagne	3.13	Italian
cosputer	computer	1/32	computer	3.13	Italian
fulm	film	1/32	film	3.13	Italian
darage	garage	1/32	garage	3.13	Italian
geseralità	generalità	1/32	generality	3.13	Italian
dapa	papà	1/32	dad	3.13	Italian
restonsabilità	responsabilità	1/32	responsability	3.13	Italian
schepa	schema	1/32	scheme	3.13	Italian
srog	strong	3/32	strong	9.38	Italian
stort	sport	1/32	sport	3.13	Italian
ubiversità	università	1/32	university	3.13	Italian
betro	berto	1/32	male name	3.13	Italian
antobus	autobus	3/29	autobus	10.33	French
clampagne	champagne	1/29	champagne	3.45	French
corfetti	confetti	2/29	confetti	6.9	French

Note: Nread indicates the number of participants in the group that read the pseudoword as real words.

Table 16. French children reading cognate pseudowords (Exp. 3)

Items	Readings	Comments
srog	(15) sʁog, (3) skʁog,<br>(2) strog, stʁog<br>(1) stoʁg, ʃʁog, ʃrog, stʁoʒ, sʁoj, sʁoʒ, sʁo	Different readings of the following graphemes<br><g> as /ʒ/, /j/ or /g/ and <s> as /s/ or /ʃ/<br>Consonant insertions between the first two letters
tigamisu	(8) tigamizy, (7) tigamisy, tigamisu<br>(2) tiʒamisu, (1) tiʒamisy, tigami	Different readings of the following graphemes:<br><g> as /ʒ/ or /g/ and <u> as /u/ or /y/<br>Syllable removal (tigami)
stort	(18) stoʁ, (4) stort, sto<br>(1) stʁo, stɔrt, stoʁʃ	Different vowel openness in <o>: /o/ or /ɔ/<br>The final consonant is either read or not
restonsabilité	(10) ʁɜstɔ̃sabilite, (4) ʁɔ̃sabilite<br>(1) ʁəstɔ̃sabili, rɜstɔ̃sabilite, ʁɜstɔ̃nasibilite	Base word reading (responsabilité - responsibility)<br>Syllable removal and addition (ʁəstɔ̃sabili - ʁɜstɔ̃nasibilite)
fratis	(13) fʁatis, fʁati<br>(4) fratis, (1) fatis	Silent final consonant pronounciation<br>Phoneme deletion (fatis).<br>Different readings of grapheme <r>: /r/ or /ʁ/
benge	(20) bɜnʒə, (5) bɜnʤ<br>(1) banʤ, bɑ̃g, bɜnʤɜ, ʒɑ̃g	Different readings of the following graphemes:<br><g> as /ʒ/,/g/ or /ʤ/ and <en> as /ɛ̃/, /ɑ̃/ or /an/
imcunité	(11) ɛ̃kynite, (4) ɑ̃kynite,<br>(1) ɑ̃komynite, impynite	Different readings for the grapheme <im>: /ɛ̃/ or /ɑ̃/<br>Base word reading (impunité - impunity)
fulm	(20) fylm, (4) fəlm, fulm<br>(1) flym	Different readings of the grapheme <u>: /y/, /u/ and /ə/<br>Phoneme inversion (flym)
corfetti	(21) kɔʁfeti,<br>(2) kɔʁf3ʁti, kɔ̃feti, kɔʁfeʁti	Base word reading (confetti)<br>Different vowel openness for <e> :/e/ or /ɜ/

Note: The numbers in parentheses indicate how many participants read the item with the following pronunciations

Table 17. Italian children reading cognate pseudowords (Exp. 3)

Items	Pronunciations	Comments
geseralità	(7) ʤɛsɛralita, (2) ʤɛnɛsalità<br>(1) ʤɛresiralita, ʤɛsarilita, ʤɛsarilita, ʤɛsalalita, ʤɛsɛrabilita,<br>ʤɛsɛlilita, kosɛralita, ʤɛseaita, ʤɛsɛrita, ʤɛnɛ:ralita, ʤjusali:ta	Pronunciations difficulties<br>Real word reading (generalità - generality)
amdio	(11) amdjo, (4) ami:do,<br>(3) ami:dɔ(2) amdi:o, ambi:o, ami:djo<br>(1) umi:dio, amɛ:djo, amo:dio, ami:djɔ, ami:djo, ambjo, ama:djo	Consonant replacements<br>Real word reading (amido - starch)<br>Different wowel lengths and openness
srog	(13) srɔg, (7) strɔg, (2) strɔng<br>(1) srɔng, trɔd, krɔ:g, snɔrd, sgrɔg<br>(1) sɔrʤ, stɔr, grɔ:g, sɔrgrɔg, sgrɔf	Pronounciation difficulties due to uncommon consonant combination (s+r)<br>Real word reading (strong)<br>Phoneme replacements, insertions, inversions, deletions.
actista	(11) akti:sta, (3) akista, (2) akti:ta<br>(1) ak:u:lista, ak:li:sta, aktista, aksi:star, aʧti:sta, aksti:sta,<br>askrita, akʧi:sta	Pronounciation difficulties due to uncommon consonant combinatio (k+t)<br>Phoneme replacements, insertions, inversions, deletions
raternità	(14) ratɛrnita, (2) ratɛrni:ta<br>(1) rateita, raternita, retarnita,reti:mrma, rantɛmita, ratɛnalita,<br>ratɛrmi:ta, ratɛminita, ratɛmita, redat:ernita, ratɛrita	Some syllables are skept<br>Tendency to retourn to a CVCV syllabic structure<br>Different vowel openness: <e>->e / textepsilon<br>Phoneme replacements or inversions
antobus	(11) antɔbus, (7) antɔ:bus<br>(5) autobus (2) anto:bus,<br>(1) autɔbus, antɔsbus	Real word reading (autobus)<br>Different vowel lengths and openness <o>as /o/ or /ɔ/<br>Phoneme insertion
orrivo	(11) or:i:vɔ, (6) ɔr:i:vɔ,<br>(4) ɔri:vɔ, ar:i:vɔ(3) ori:vɔ,<br>(1) o:r:ivo, aor:i:vɔ	Real word reading (arrivo - arrival)<br>Different vowel lengths and openness <o>as /o/ or /ɔ/
imcunità	(13) iŋkunita, (4) iŋkomunita<br>(1) iŋknunita, iŋkuita, im:unkita, imsunita,<br>iŋgunita, iŋku:nita, komunita, iŋkumita, im:unita	Real word reading (immunità - immunity)<br>Real word reading (comunità - community)<br>Phoneme insertions, deletions and replacements
restonsabilità	(11) rɛstɔnsabilita, (3) rɛspɔnsabilita,<br>(2) rɛstɔnabilita, (1) rɛstɔrnsi:ba, rɛzostambilita, rɛstaubalita,<br>rɛstɔbilita, rɛstɔnsibilita, rɛstɔnansabilita, rɛstɔlisabilita	Real word reading (responsabilità - responsability)<br>Addition or removal of syllables and phonemes<br>Different readings for the grapheme <s>: /s/ or /z/
alsterità	(11) alstɛrita, (6) alsɛrita<br>(1) altrɛri:sta, altɛsenita, alstɛrilita, alstɛralita,<br>altɛri:sta, alastralita, alstɛrnita, alsɛita	Removal of syllables and phonemes<br>Consonant cluster simplification by addying vowels

Note: The numbers in parentheses indicate how many participants read the item with the following pronunciations

Table 18. List of pseudowords read as real words in Experiment 4

nonword	word	nread	percentage	participants
asiet	aside	1/38	2.62	English
deorly	dearly	3/38	7.9	English
dise	dice	4/38	10.53	English

Note: Nread indicates the number of participants in the group that read the pseudoword as real words.

Table 19. German adults reading frequency matched pseudowords (Exp. 4)

Items	Pronunciations	Comments
quaw	(6) kva:f, (2) kua:,<br>(1) ka:, kuab, kuaf, kva:, kva:v	Different readings for the following graphemes:<br><u>as /v/ or /u/ and <w>as /v/, /f/, /b/ or ø<br>Different vowel lenths + Phoneme deletion (ka:)
spafe	(9) ʃpa:fe, (4) spa:fe,<br>(1) ʃpafe, spa:ve	Different readings for the following graphemes:<br><s[p]>as /ʃ/ or /s/ and <f> as /f/ or /v/<br>Different vowel lengths
jotzt	(11) jotst,<br>(1) jøtst, jots, jo:tst, jost	Different readings for the grapheme <o>: /ø/ or /o/<br>Different vowel lengths + Phoneme inversions (jost)
hog	(10) ho:k,<br>(3) hok, (2) hog	The final consonant devoicing rule is either applied or not<br>Different vowel lengths
mab	(9) ma:p,<br>(5) map, (1) mab	The final consonant devoicing rule is either applied or not<br>Different vowel lengths
stenn	(11) ʃten,<br>(2) sten, (1) ʃte:n	Different readings for the grapheme <s>: /ʃ/ or /s/<br>Different vowel lengths
peren	(13) pe:ren, (1) pe:re:n, peren	Different vowel lengths
mat	(7) ma:t, (6) mat	Different vowel lengths
üfrer	(8) y:frer, (7) yfrer	Different vowel lengths
gerielst	(10) geri:lst<br>(2) ge:ri:lst, (1) ge:rilt	Different vowel lenghts<br>Phoneme deletion (ge:rilt)

Note: The numbers in parentheses indicate how many participants read the item with the following pronunciations

Table 20. German adults reading dissimilar pseudowords (Exp. 4)

Items	Pronunciations	Comments
vitifu	(6) viti:fu, (3) vi:tifu,<br>(2) fitifu, fi:ti:fu,<br>(1) fiti:fu	Different readings for the grapheme <v>: /v/ or /f/<br>(in real word reading it would be /f/)<br>Different vowel lengths
zigidu	(8) tsigidu:, (5) tsigi:du,<br>(1) zigidu:, zi:gidu	Different readings for the grapheme <z>: /ts/ or /z/<br>Different vowel lengths
hevimi	(10) he:vi:mi,<br>(3) he:vi:mi:,<br>(1) he:vimi, he:fi:mi	Different readings for the grapheme <v>: /v/ or /f/<br>(in real word reading it would be /f/)<br>Different vowel lengths
zulumu	(8) tsulu:mu, (2) tsulumu:<br>(3) tsu:lumu, (1) zulumu:, zu:lu:mu	Different readings for the grapheme <z>: /ts/ or /z/<br>Different vowel lengths
ledigi	(7) le:digi, (5) le:di:gi,<br>(1) le:digi:, ledigi, legi:gi	Different vowel lengths<br>Phoneme replacement (legi:gi)
lopove	(9) lo:po:ve,<br>(2) lopo:fe, lo:po:fe,<br>(1) lopofe, lo:po:ve:	Different readings for the grapheme <v>: /v/ or /f/<br>(in real word reading it would be /f/)<br>Different vowel lengths
luxeto	(10) lukseto,<br>(4) lukse:to, (1) luksedo	Different readings for the grapheme <t>: /t/ or /d/<br>Different vowel lengths
tizafe	(10) ti:tsa:fe, (4) ti:tsafe,<br>(1) ti:za:fe	Different readings for the grapheme <z>: /ts/ or /z/<br>Different vowel lengths
rimuze	(7) ri:mu:tse, (7) rimu:tse,<br>(1) ri:mu:tse:	Different vowel lengths
galido	(11) gali:do, (2) ga:li:do,<br>(1) ga:lido:, galido:	Different vowel lengths

Note: The numbers in parentheses indicate how many participants read the item with the following pronunciations

Table 21. French adults reading frequency matched pseudowords (Exp. 4)

Items	Pronunciations	Comments
regils	(11) ʁəʒil, (2) regils, ʁəglis<br>(1) ʁegil, ʁeʒis, ʁəglils, ʁəʒəli, ʁə	Different readings for the graphemes:<br><g>: /g/ or /ʒ/, <e>: /e/ or /ə/ and <r>: /r/ or /ʁ/<br>The final consonant is either read or not
fremd	(11) fʁɑ̃, (8) fʁɛ̃d,<br>(3) fʁɑ̃d,(1) bʁɛ̃d	Different readings for the grapheme <em>: /ɑ̃/ or /ɛ̃/<br>Phoneme replacement f -> b and deletion (fʁɑ̃)
brend	(12) bʁɛ̃d, (9) bʁɑ̃<br>(4) bʁɑ̃d, (2) bʁɛ:n, (1) bʁɛ̃	Different readings for the grapheme <en>: /ɑ̃/, /ɛ̃/ or /ɛ:n/<br>The final consonant is either read or not
etcere	(18) ɛtsɛʁ, (5) ɛsɛʁ<br>(1) etsɛʁ, ɛtsœʁ, ɛtsɛʁə, ɛts, ɛtsɛtʁ	Different readings for the grapheme <e>: /œ/, /ɛ/, /e/ or /ə/<br>The final <e> is either read or not<br>Phonemes deletions (ɛts, ɛtsɛʁ) or insertion (ɛtsɛtʁ)
degias	(19) deʒja, (5) deʒjas<br>(1) degias, deja	Different readings for the grapheme <g>: /ʒ/, /g/ or //<br>The final consonant is either read or not
panbing	(21) pɑ̃biŋ, (3) pɑ̃bɛ̃ŋ<br>(2) pɑ̃bɛ̃, (1) pɑ̃bin, pɛ̃bɛ̃	Different readings for the graphemes:<br><an>: /ɑ̃/ or /ɛ̃/, <in>: /in/, /iŋ/ or /ɛ̃/ and <n>: /n/ or /ŋ/
casment	(21) kasmɑ̃, (4) kazmɑ̃,<br>(1) kasmɛ̃t, kasmɑ̃t, ka	Different readings for the graphemes:<br><en>: /ɑ̃/ or /ɛ̃/ and <s>: /s/ or /z/<br>The final consonant is either read or not
fleuiller	(20) flœje, (6) fœje<br>(1) flœle, fyøle	Real words readings:<br>feuiller - come into leaf & fiole - phial<br>Different readings of the grapheme <ill>: /j/ or /l/
tausait	(19) tozɛ, (7) tosɛ<br>(2) tose	Different readings for the graphemes:<br><e>: /ɛ/ or /e/ and <s>: /s/ or /z/
duntre	(23) dœ̃tʁ, (2) dyntʁ<br>(1) kuntʁ, dœ̃, dɑ̃tʁ	Different readings for the grapheme <un> : /œ̃/, /yn/, /un/. /ɑ̃/<br>Phoneme replacement /d/ -> /k/

Note: The numbers in parentheses indicate how many participants read the item with the following pronunciations

Table 22. French Adults reading dissimilar pseudowords (Exp. 4)

Items	Pronunciations	Comments
tesusa	(8) tesusa, (7) tezyza, (3) təzyza,<br>(1) tesyza, tesysa, tysusa, tezuza, tezyz	Different readings for the graphemes:<br><s>: /s or /z/; <u>: /u/ or /y/ and <e>: /e/ or /ə/<br>Phoneme deletion (tezyz)
ledigi	(10) lədiʒi, (11) lediʒi,<br>(5) ledigi, (1) legiʒi, lədigi	Different readings for the graphemes:<br><g>: /g/ or /ʒ/ and <e>: /e/ or /ə/<br>Phoneme replacement
buleba	(14) byleba, (7) byləba, (3) bylba,<br>(2) buleba, (1) belyla, pyleba	Different readings for the graphemes:<br><u>: /u/ or /y/ and <e>: /e/ or /ə/<br>Phoneme deletion (bylba) and devoicing /b/ ->/p/
zigidu	(13) zigidy, (9) ziʒidy,<br>(4) zigidu, (1) ziʒidu, ziʒibyty	Different readings for the graphemes:<br><g>: /g/ or /ʒ/ and <u>/u/ or /y/<br>Addition of syllable (ziʤibyty)
gobujo	(21) gɔbyʒo, (1) gɔbuʤo, gɔbyʒœ, gɔbyʒy,<br>bɔʒyʒy, gɔbyjʤo, gogɔbuʒo, go	Different readings for the graphemes:<br><g>: /g/, /ʒ/ or /ʤ/, <u>/u/ or /y/ and <o>: /o/ or /ɔ/<br>Addition of syllable (gogɔbuʒo) + Mispronunciation (go)
luxeto	(21) lykseto, (5) lyksəto<br>(1) lyksito, lykzeto	Different readings for the graphemes:<br><x>: /ks/ or /kz/ and <e>: /e/, /i/ or /ə/
fuduja	(24) fydyʒa<br>(1) fuduʤa, fedyʤa, fydja, fydyʤa	Different readings for the graphemes:<br><g>: /j/, /ʒ/ or /ʤ/ and <u>/u/ or /y/<br>Phoneme replacement /u/ ->/e/ and deletion (fydja)
zulumu	(24), zylymy, (3) zulumu<br>(1) zybymy	Different readings for the grapheme <u>: /u/ or /y/<br>Phoneme replacement /l/ ->/b/
mumade	(25) mymad,<br>(1) mumad, mumade, myman	Different readings for the grapheme <u>: /u/ or /y/<br>The final <e>is either read or not<br>Phoneme replacement /d/ ->/n/
vitifu	(25) vitify, (2) vitivy, vitifu<br>(1) fitiflu	Different readings for the grapheme <u>: /u/ or /y/<br>Consonant assimilation and Phoneme insertion (fitiflu)

Note: The numbers in parentheses indicate how many participants read the item with the following pronunciations

Table 23. English adults reading frequency matched pseudowords (Exp. 4)

Items	Pronunciations	Comments
asiet	(13) asiet,<br>(1) ast, asit, asite, assist, aset, iset	Different readings for the grapheme <ie>: /i/ or /e/<br>Phonemes deletion (ast), replacement (a -> i)<br>Real word reading (assist)
aecrer	(4) aesrer, (2) aser, asra, (3) asrer,<br>(1) ases, aseri, aeksrer, asresser, asekrer, aserer, askrer	Phonemes and syllables deletions (ases),<br>and phoneme insertions (asekrer, asresser)
strylture	(10) strailture, (4) strailiture,<br>(1) strai, straiIetto, straili, straiturle	Phonemes and syllables addition (strailiture),<br>deletions (strai) and inversions (straiturle)
watheet	(9) wai:t, (4) wati:t,<br>(1) wahti:st, waet, wati:, atpi:t	Different readings for the following graphemes:<br><ee> as /i:/ or /e/ and <th> as /t/ or //
dousse	(13) dousse, (4) dosse,<br>(1) doussi:s, deuse	Different readings for the following graphemes:<br><ou> as /ou/, /o/ or /eu/ and <e> as /e/ or /i/
deorly	(17) deorli,<br>(1) dori, dearli	Different readings for the grapheme <eo>: /eo/, /ea/ or /o/<br>Phoneme deletions (dori)
rebube	(16) rebube,<br>(2) rebubi:, (1) rubab	Different readings for the grapheme <e>: /e/ or /i:/<br>Phoneme deletions (rubab) and relacements (e -> u; u -> a)
dise	(10) dise, (4) diese,<br>(3) diss (1) daese	Different readings for the grapheme <i>: /i/, /ie/ and /ae/<br>Phoneme deletion and consonant doubling (diss)
speached	(18) spi:ched,<br>(1) spleched	Different readings for the grapheme <ea>: /i/ or /e/<br>Phoneme addition (spleched)
vuing	(15) vuing,<br>(1) voning, vling, vigged	Different readings for the grapheme <u>: /u/ or /o/<br>Phonemes addition (voning, vling, vigged)

Note: The numbers in parentheses indicate how many participants read the item with the following pronunciations

Table 24. English adults reading dissimilar pseudowords (Exp. 4)

Items	Pronunciations	Comments
buleba	(10) buleba, (1) buleaba, bubala, blubabd, bulba, bulejub, bubela, buliba, beleba	Phonemes deletions (bulba), insertions (blubabd) and inversions (bubela, bubala)
tizafe	(10) tizafe, (5) tizaf, (1) tizaife, zafafe, tizave	Different readings for the grapheme <f>: /f/ or /v/ Phoneme deletion (tizaf, zafafe)
ledigi	(18) ledigi, (1) ledgili, ledgi	Phonemes deletion (ledgi) and addition (ledgili)
gobujo	(14) gobujo, (1) goboju, gobuju, goguba	Different readings for the grapheme <o>: /o/ or /u/ Syllable reduplication (goguba)
luxeto	(12) lukseto, (4) luksetto, (1) leksuto, luzejto	Consonants doubling (luksetto) Phonemes replacements (e ->u; ks ->z)
rimuze	(7) rimuse, (4) rimusi, (1) rimuzu, remuse, rismuse	Different readings for the following graphemes: <e> as /i/ or /e/ and <z> as /z/ or /s/ Phoneme addition (rismuse) and replacement (u -> e)
pazile	(9) pazille, (7) pazile, (1) pazit	Consonant doubling (pazille) Phoneme deletion and replacement (pazit)
zigidu	(13) zigidu, (1) zigido, zizidu, zigudu, zigidiu, zikidi	Different readings for the following graphemes: <u> as /u/ or /o/ and <g> as /g/ or /k/ Syllable reduplication (zizidu) and vowels assimilation (zikidi)
zulumu	(15) zulumu, (1) zumulu, zlumu, zulumi	Syllables inversion (zumulu) Phoneme deletion (zlumu) and replacement (u ->i)
hevimi	(14) hevimi, (1) hemivi, hevim, hevilimi, hevini	Syllables inversion (hemivi) and addition (hevilimi) Phoneme deletion (hevim) and replacement (m ->n)

Note: The numbers in parentheses indicate how many participants read the item with the following pronunciations
